# The Soviet doctor and the treatment of drug addiction: "A difficult and most ungracious task"

**DOI:** 10.1186/1477-7517-8-32

**Published:** 2011-12-30

**Authors:** Alisher B Latypov

**Affiliations:** 1 Eurasian Harm Reduction Network (EHRN), 11B Svitrigailos Street, Vilnius 03228, Lithuania; 2 Global Health Research Center of Central Asia (GHRCCA), Columbia University, 1255 Amsterdam Avenue, New York, NY 10027, USA

**Keywords:** Soviet narcology, history, social and mental hygiene, psychiatry, addiction treatment, opiate maintenance therapy, repression of drug users

## Abstract

This paper reviews the development of early Soviet drug treatment approaches by focusing on the struggle for disciplinary power between leading social and mental hygienists and clinical psychiatrists as a defining moment for Soviet drug treatment speciality that became known as "narcology." From this vantage point, I engage in the examination of the rise and fall of various treatment methods and conceptualizations of addiction in Russian metropolitan centres and look at how they were imported (or not) to other Soviet republics. As clinical psychiatrists appeared as undisputed victors from the battle with social and mental hygienists, the entire narcological arsenal was subdued in order to serve the needs of mainstream psychiatry. However, what that 'mainstream' would be, was not entirely clear. When, in 1934, Aleksandr Rapoport insisted on the need for re-working narcological knowledge in line with the Marxist approach, he could only raise questions and recognise that there were almost no "dialectically illuminated scientific data" to address these questions. The maintenance treatment of opiate users, which emerged as the most effective one based on the results of a six-year study published in 1936, was definitely not attuned to the political and ideological environment of the late 1930s. Maintenance was rather considered as a temporary solution, in the absence of radical therapeutic measures to free Soviet society from "narkomania." As the Great Terror swept across the Soviet Union, Stalin's regime achieved its objective of eliminating drug addiction from the surface of public life by driving opiate users deep underground and incarcerating many of them in prisons and the Gulag camps. In the final section, I briefly discuss the changing perceptions of drug use during the World War II and outline subsequent transformations in Soviet responses to the post-war opiate addiction [Additional file 1].

## 

When in 1922 a prominent Central Asian psychiatrist, Leonid Antsyferov, presented his paper on "hashishism in Turkestan" at the First Scientific Congress of Physicians of Turkestan, he called for the Soviet government to take a number of urgent measures aimed at rooting out this 'evil' or, if such an objective would prove unrealistic in a short term, then at least at reducing it to its smallest possible extent. He argued that repressive measures alone would be insufficient and that the struggle against drug addiction should be conducted by different means, namely enlightenment and treatment. In his view, physicians were to be placed at the head of this struggle because the task of making the masses healthier was particularly close to their mandate [1]. However, at the heart of these anti-drug activities were questions to which Antsyferov did not submit straightforward answers: who would be responsible for treating drug addiction and how these specialists would need to carry out their job?

### The rise and fall of *narkodispanser*

These questions were surely not new to biomedical doctors, who were used to dealing with the opiate consumer both in the heartland of the Russian Empire and in Russian Turkestan. Together with chronic alcoholics and consumers of other drugs, opiate addicts were believed to be in need of treatment and the authority for their treatment belonged to psychiatrists. Well before the Bolshevik's revolt, in the second half of the nineteenth century, Russian physicians began to publish their works on opiates and opiate addiction. Some of them, including pre-eminent psychiatrist from Moscow, Sergei Korsakov (1854-1900), discussed the use of opium in psychiatry and stressed that opium could be a very useful medicine that could be administered in the form of a powder, a pill or an enema for controlling a wide range of conditions including agitated boredom, manic excitement, delirium tremens and epilepsy [2,3]. Other physicians wrote about the potency of opium, the way in which it could alter the consciousness of humans, and the treatment of morphine and alcohol addicts with hypnosis [4,5]. When in December 1902 P. Ortenberg arrived at Palassan village following a major earthquake in Andizhan District of the Russian Military General Governorship of Turkestan, he came across with local *kuknar *(a liquor made by soaking in water the dried and bruised capsules of the poppy) users. He then engaged in treating them with valerian drops and by convincing every patient on the "enormous harms self-inflicted by the use of *kuknar*" - a method which he called 'the psychiatric persuasion' [6]. Although special establishments for the treatment of drug addicts did not exist in pre-revolutionary Russia and its colonial dominions, there was a general agreement among physicians that, as far as 'morphinists' were concerned, such treatment should take place within an in-patient unit, under close supervision of doctors and nurses. Very often, such facility was located within the walls of a psychiatric asylum. The three most common methods used to treat 'morphinism' in Russia were guided by late nineteenth century works written by German authors. According to one contemporary Russian psychiatrist, they were fundamentally different from each other in one major aspect, and that was the decision of whether to withdraw the drug from a patient (i) abruptly, according to Dr Eduard Levinstein's method, (ii) rapidly, within one to two weeks after admission, according to Dr Albrecht Erlenmeyer's method or (iii) gradually, within one to two months, in line with Dr Rudolph Burkart's method [7].^a^

The First World War, the Bolsheviks' revolution and subsequent Civil War in Russia sent Russian psychiatry into disarray and existing mental hospitals to the verge of collapse. As described by Irina Sirotkina, the death rate in psychiatric hospitals reached forty percent during the Civil War. For many months, psychiatric facilities were left without any fuel, food and drug supplies, with both patients and staff starving. As a 1920 survey of forty-eight psychiatric hospitals has shown, more than half of these institutions faced heavy shortages of food supplies. Infectious diseases were rampant and in winter times some facilities were not in a position to maintain the indoor temperature above freezing point [8]. For many psychiatrists, collaboration with the new regime was seen as the only way to address this disastrous situation in Russian psychiatry.

In June 1918, at the First All-Russian Congress of Medical-Sanitary Sections, the future People's Commissar of Public Health, Nikolai Semashko (1874-1949), outlined the new principles of Soviet medicine. In the Soviet Union, the treatment was to be provided free of charge within a unified system, which would place a heavier emphasis on prevention through sanitation and other social measures [9]. Semashko became the leading proponent of social medicine and the chief patron of social hygiene, which he defined as "the science of the influence of the economic and social conditions of life on the health of the population and on the means to improve that health" [[10]: p255]. Semashko actively supported the German-Russian links in social medicine and Soviet social hygienists were generally willing to openly acknowledge their intellectual debt to German *soziale Medizin *[11]. Among those physicians who wholeheartedly shared the plans of the Soviet People's Commissariat of Public (Narkomzdrav) to prioritize a preventative approach and pay particular attention to socio-economic determinants of health was the future architect of the Soviet mental health care system, psychiatrist Lev Markovich Rozenshtein (1884-1934).

Rozenshtein joined Narkomzdrav soon after it was established. He became an active member of its Neuro-Psychiatric Section (originally established as a Psychiatric Commission), raising his voice against the old and therapeutically sterile "custodial psychiatry" ("*psikhiatriia prizreniia*") and supporting "active psychiatry" [8,12]. As early as 1914, Rozenshtein began to use the model of dispensary care for alcoholic and nervously ill patients as he opened a community-based outpatient facility in a factory village near Moscow. Central to this approach was the idea of social work in the community with a major goal of prevention of mental illnesses, - the idea communicated by the Swiss-American psychiatrist Adolf Meyer at the International Congress of Medicine in London in 1913, which Rozenshtein attended together with a group of fellow compatriots at the end of their study tour to European psychiatric services [8,12].

In a paper given in London, "The Aims of a Psychiatric Clinic", Adolf Meyer only briefly referred to a dispensary as a community centre that deals "with that type of case that might by no means necessarily be willing to consider himself, or would not be considered by others, as sufficiently disturbed mentally to require removal to a state hospital or asylum." Nevertheless, he declared that the modern spirit of medicine demanded both the work of prevention and of cure, with the close relationships between social work and the psychiatric hospital serving as "one of the fundamental and most important factors of progress in psychiatry." "Social service, as we call this work in the United States," Meyer remarked, "is the agency which reaches into the home and makes it its duty to supervise the conditions outside of the hospital and the activity of the patient in relation with the family and the community." "...[I]n shaping a clinic or a hospital for model work it becomes essential that it should be planned so that the social work as well as the study of individual case...should be as practicable and effective as possible" [[13]: pp363-367]. Eighteen years later, in his article entitled "Public Health Service and Mental Hygiene in the USSR," Rozenshtein maintained this connection between his reformist activities in Soviet Russia and Meyer's influence on him by stating as follows: "In 1913, as a member of the International Congress of Physicians in London, I was present at a discussion between Adolf Meyer and the Scotch psychiatrists as to the best method of developing psychiatry. History has shown the correctness of the view of Adolf Meyer and American psychiatry" [[8]: p230, [14]].

Yet, during the initial years after the revolution and in the early 1920s, many Russian psychiatrists did not accept the idea of preventive psychiatry and mental hygiene as enthusiastically as Rozenshtein did. In 1919, despite the explicit appeal for increased focus on the prevention of mental illness made by the deputy Commissar of Public Health in his opening speech at the First All-Russian Neuropsychiatric Conference (*soveschanie*), the delegates advocated for the restoration of mental colonies as well as for the prioritization of care of chronic patients over acute ones. While some of them viewed this call unrealistic because of the on-going Civil War in the country, others did not want to change the 'traditional' system and the asylum to play an inferior role [15]. For those traditionally-minded asylum psychiatrists, narkomania (drug addiction) appeared as a mental illness that often had to be treated on a compulsory basis in a psychiatric hospital or colony while narkomans (drug addicts) were described as "morally depraved" "psycho- and neuropaths" and "degenerates." As asserted in 1921 by the author of one of the earliest Soviet review articles on "narkomania," M. P. Kutanin, drug addiction apparently had a very severe prognosis. Treating the addict in a general clinic or at his home, in the community and without strict control and supervision, was believed to "lead to nothing," thus making the treatment of narkomania, in his view, "a difficult and most ungracious task" for the psychiatrist [[16]: pp36-51].

However, as historian Susan Gross Solomon emphasises, the commitment to social medicine and social hygiene was of fundamental importance within Narkomzdrav. With the support from the Commissar of Public Health himself, the formal process of institutionalization of social hygiene in Soviet public health was set in motion by 1922 through the opening of the first department of social hygiene in Moscow, the introduction of the subject of social hygiene into the university curricula and the launch of a new journal, *Sotsial'naia gigiena*, dedicated to social hygiene research. These developments were followed by the establishment of the State Institute for Social Hygiene in 1923 [8,11]. Rozenshtein, too, had acquired considerable power since the time of the first conference of Russian neuropsychiatrists by combining the advancement of his academic research activities with top level professional work in Narkomzdrav and in the major Bolshevik associations [8]. In November 1923, at the Second All-Russian Conference on the Questions of Psychiatry and Neurology, Rozenshtein and his colleagues (Zinov'ev, Prozorov, Strashun and Sysin) were ready to draw the attention of all participants to the new tasks of Soviet psychiatry, which included the organization of neuro-psychiatric dispensaries as the primary institution for the Soviet mental health care system. As Rozenshtein and his ally P. M. Zinov'ev proposed, these dispensaries were now to become "the organs of applied psychology"; centres for the provision of outpatient care and "the catchment" of the mentally and nervously ill at the early stages of disease - the cases in which "the disease did not yet damage the working capacity of the organism"; and social welfare, research, sanitary enlightenment and counselling institutions functioning in close contact with the working masses [[8,17]: pp73-74]. Among those who believed in feasibility of this ambitious project, was psychiatrist Aleksandr Sholomovich. He had apparently broken his ties with traditional psychiatry in the wake of the Bolshevik revolution and was now raising the question of organizing specialized narcological dispensaries following an initial experience of placing a 'physician-narcologist' and a nurse trained in provision of social care at five Moscow-based dispensaries for the tubercular [10,17,18].

The following year, with the back up from Narkomzdrav, both Rozenshtein and Sholomovich were celebrating their first victories as the first Soviet neuro-psychiatric dispensary became operational at the Moscow Psycho-Neurological Institute and the first narcological dispensary, or *narkodispanser*, was opened at the former *Miasnitskaia *Hospital [8,18]. In May 1925 Rozenshtein's institution was elevated to the status of independent Moscow State Neuro-Psychiatric Dispensary, with funding coming from the centralised state budget and with the mandate to provide methodological guidance to the process of organizing psychiatric care throughout Russia. Rozenshtein could now oversee what Sirotkina, paraphrasing Lenin, described as "dispensarization" of the entire country [8]. In the words of Rozenshtein's ally, Zinov'ev, this was to be achieved through the efforts of mental hygiene specialists by "searching out the mentally ill and those...in danger of mental illness" [[8]: p157, [19]]. As for Sholomovich, he now had new and important tasks lying ahead of him as he had to reshape narcology and to prove that theirs was the right approach. Sholomovich's confidence must have been further boosted when the Public Health Commissar joined the Moscow Narcological Society (formed in 1927), in the capacity of the head of organisational committee.

In 1926, when the first volume of *Issues of Narcology *was published under the editorship of Sholomovich, he began from the very basics and opened the volume with the discussion of terminology and key notions of the "*narkoproblema*." He immediately drew a connection between social hygiene and narcology by referring to drug specialists as "social hygienists-narcologists." His criticism of psychiatry was even more evident when he elaborated on the drug use phenomenon. While suggesting that the notion of "narcosis" was fundamental to understanding the drugs issue, Sholomovich refused to apply the term "narkomania" for the purposes of an indiscriminate explanation of different forms and patterns of drug use. In his view, "mania" effectively implied the presence of some sort of 'pathology' in an individual, and, when used by psychiatrists in their clinical research, the term "narkomania" invoked the image of a psychotic patient with the underlying problem of "poisonous" substance abuse. These 'narcotic poisons' also included alcohol. However, for masses of people who were "moderate" consumers of 'narcotic poisons' and never ended up as 'psychotics' inside the walls of the psychiatric asylum, using the term "narkomania", which belonged to the realms of psychiatry, was unacceptable. These people, Sholomovich argued, could not be called "narkomans", a shortened version for narcomaniacs, and therefore a different term was needed. Such a term that could be employed to refer to the drug use phenomenon in 'real life' (*termin, primenimyi v zhizni*) was, in his view, "narkotism" [[20]: pp5-7].^b^

Sholomovich continued his crusade against traditional psychiatry in two other essays published on the pages of the same volume. In one of these papers he argued that "one could not eliminate the consequence [narkotism] without doing away with the causes," which were social and economic in their nature. It was not a psychiatric hospital but rather *narkodispanser *that he advocated as the best tool for prevention and treatment of social diseases. An enlightenment project in its essence, *narkodispanser *would "enter the life of narkoman" as a "caring friend" and "authoritative counselor" and "illuminate, with such a bright light, both the entire everyday life [*byt*] of the patient and the source of his disease..." [[21]: pp45-50].

He finished by openly declaring "the break-off with traditional psychiatry" and admitting that this had been the most difficult task facing the Moscow-based Narkosection - "the first and so far the only Soviet squad to fight narkotism." How did he explain this 'separation' of narcology from psychiatry? Narkomans with psychiatric co-morbidity (*dushevno-bol'nye narkomany*), he claimed, represented a minute proportion of the entire population, equaling 0.002 percent. This was only a drop in "the ocean of habitual [*bytovoi*], massive everyday life narkotism," which included alcohol use and in his view affected as much as 70 to 80 percent of the adult male population. The psychiatrist, with his "therapeutic spoon" would never be able to deplete this ocean of "social pathology" and, therefore,

*Indebted to psychiatry for its origin, narcology (this is how we call the entire, broad area of issues connected with the problem of narcotic poisons), having grown up, detaches itself from the maternal soil and enters...a new, independent pathway*...[[18]: p72].

As Sholomovich believed, the above process of "natural evolution" was common in science.

The timing of Sholomovich's attack on psychiatry could not be more fortunate for a cohort of leading Soviet social hygienists, who were also entering the battle with psychiatrists for the hegemony over habitual alcoholism research and treatment. On 11 September 1926, with reports of apparently rising alcoholism appearing in the press, the Council of People's Commissars (Sovnarkom) issued a decree on immediate measures in the field of the struggle against alcoholism and charged Narkomzdrav with "strengthening systematic research" on alcoholism and the prophylactic fight against it. In February 1927, Narkomzdrav, headed by Semashko, issued a circular passing the responsibility for the systematic study of alcoholism as a social disease to the State Institute for Social Hygiene [10,22-24]. With the leading advocate and mastermind of *narkodispanser *announcing the independence of 'daughter-narcology' from its 'mother-psychiatry' and the marriage between narcologists and social hygienists, it should not be surprising that *narkodispanser *came to be viewed by many as one of the strongholds of social hygiene.

What were the key principles of the unique approach of *narkordispanser *to narkotism? Firstly, the strong emphasis on causes rather than consequences clearly implied that prevention would be given higher priority over treatment. According to Sholomovich, once poverty, the absence of human rights and economic exploitation common to capitalism were addressed, people's reliance on 'narcosis' and consumption of all kinds of 'narcotic poisons' in order to achieve harmony was eventually to be replaced with "physiological cultural narcosis." Under the new rule of the Bolsheviks, Soviet citizens would be "inebriated by culture, science, the beauty and harmony of life and labour" [[21]: pp45-47]. Secondly, by shifting their focus from 'mania' to 'ism' narcologists were also suggesting that no matter what type of treatment they provide, it would be different from the long-term residential or custodial care of traditional psychiatrists. As soon as Sholomovich began to publish his articles on the 'struggle against narkotism' and to report on the activities of *narkodispansers*, one of his main tasks was to concentrate on how the above principles were translated into concrete results in 'real life'.

Propaganda against the 'dope' (which included all types of 'poisons' including alcohol) was considered as one of the main activities of the Narkosection and was usually performed through lectures, talks, meetings and actual and mock trials of 'narkomans', subjecting them to public censure. Overall, Sholomovich claimed, the Moscow Narkosection specialists delivered 805 lectures by the end of the first quarter of 1925, covering as many as 70,000 workers and students [18]. Although the number of new patients (who included not only alcohol drinkers and users of other 'poisons' but also their family members with neurosis, which they developed as a result of the presence of the '*narkoproblema' *in the family) seen in Moscow between 1924 and 1926 was not as high and totaled 16,500 people, Sholomovich proudly stated that these clients were handled by as few as 2 *narkodispansers *and 6 *narkopoints *(*narkopunkty*) [25].

However, as far as the treatment was concerned, he had to admit that this 'problem' was far from its resolution, with the methods of drug treatment being at the very early stages of their development [18]. One of such new, 'active' methods of treatment introduced by the Moscow-based narcologists was subcutaneous oxygen therapy, or the so called '*O-terapiia*'. Although initial experiments with subcutaneous injections of oxygen were conducted as early as the 1770s, it seems to have been first used for the therapeutic purposes in 1799 by Thomas Beddoes at the Pneumatic Institute in Bristol, UK. In the 1910s and 1920s, as a result of research conducted in Europe and North America and aimed at developing a better understanding of the effectiveness of supplemental oxygen and finding an effective way of oxygen administration to the patient, the use of subcutaneous oxygen therapy increased significantly for a wide range of indications [26]. In Russia, Sholomovich advocated subcutaneous '*O-terapiia*' primarily for the treatment of 'morphinism', although he reported that some 'cocainists' and alcoholics received it as well. According to Sholomovich, subcutaneous oxygen therapy was introduced for the purposes of treatment of morphinism because it was proved to provide an equivalent degree of euphoria [18].

It seems more likely that, similar to the case of Rozenshtein's 'model' neuro-psychiatric dispensary, Moscow-based *narkodispanser *received its equipment for subcutaneous oxygen therapy from abroad at a later stage and therefore had only a very limited experience of its administration to patients. Thus, out of about 585 'morphinists' seen by the Moscow Narkosection between 1924 and the first nine months of 1925 (5-7 percent out of the total of 8370 new patients)^c ^only 21 patients were reported to have been administered *O-terapiia *subcutaneously. Nevertheless, Sholomovich asserted that all of these patients were 'greatly delighted' with oxygen therapy and provided very positive feedback. *O-terapiia *supposedly made it very easy for all morphinists, who received this type of treatment, to substantially reduce their daily doses of morphine, with many patients going down from 1.5 to 0.05 (unspecified units) of pure morphine without experiencing abstinence symptoms [18].^d^

Despite this initial success of the new treatment method, on its own, it seemed to be insufficient to 'entirely eliminate' morphinism and, as one of patients commented in his letter to *narkodispanser*, "...for the ultimate cure of the weakened will of a morphinist, something else is needed." It was removing the patient from his social milieu and placing him to an in-patient unit, or *statsionar*, for a period of two to four weeks that would have to become the ultimate fix, as Sholomovich admitted [[18]: p77]. However, given the absence of such in-patient units at the majority of *narkodispansers *as well as at every *narkopoint*, the only thing that narcologists were often able to do for morphine and cocaine users was to limit their intervention to the provision of advice [25]. Leading to disillusion among the clients, in effect, this also meant that many of the patients would eventually have to be dealt with in a 'traditional' psychiatric establishment.^e ^Although Sholomovich himself reported that only 7 to 10 percent of his patients had to be referred to psychiatric hospitals and that even a smaller proportion of 1 to 2 percent of patients were 'psychopaths' who supposedly belonged to a psychiatric clinic, other psychiatrists who supported the idea of *dispanser *and its "apparatus for social assistance" were far less optimistic. In Leningrad, for instance, Professor Raisa Ia. Golant suggested that out of 189 patients with opiate addiction treated in the *dispanser*, 22 percent of males and 12 percent of females were, "undoubtedly," psychopaths. In another example, A. N. Kondratchenko, who performed an out-patient observation of 162 opiate addicts between 1927 and 1929 in Tashkent and acknowledged Sholomovich's contribution in developing the theme of his article, similarly described as many as 48 'opiomans' from his sample as 'psychopaths'[18,27,28]. By 1928, psychiatrists began to report that increasing number of patients with a history of (unsuccessful) treatment at *narkodispanser *were showing up at residential psychiatric institutions [10]. Indeed, there seemed to have been a certain irony about the effectiveness of *dispanser *even among non-specialists, as common people were singing songs about *dispanser *being more likely to fail in curing an alcoholic [29].

However, in order to have a more complete account on the sustainability of the *narkodispanser *project, challenges related to the treatment of 'narkotists' (as Sholomovich often referred to alcohol and drug users without 'manias') also need to be put in the context of social hygienists' research on alcoholism that appeared after 1926.

The questionnaire that was developed by the Director of the State Institute for Social Hygiene, A. V. Mol'kov, was focused on 'exogenous' (life-style) factors contributing to habitual (*bytovoi*) alcoholism and was widely replicated in different surveys on the local level, covering no less than 40,000 respondents [10,24]. As Solomon highlights, post-revolutionary Soviet culture showed a remarkable commitment to 'science', with questionnaires carrying with them "an aura of science." For social hygienists, the use of such instruments helped to strengthen their claims to legitimacy [[11]: p187]. This does not mean, though, that these questionnaires were methodologically unproblematic and, as this was the case with Mol'kovs device, it was heavily biased towards showing the importance of life-style, habit, education and culture in alcohol use. When used in Uzbekistan by local social hygiene specialists and students at the forefront of the struggle against alcoholism, these questionnaires, designed for collecting data from "all conscientious citizens" in order to draw conclusions on the ways of fighting against alcoholism, were further elaborated to include the names of local intoxicants such as "buza", "musalias", and even "kumys" produced from mare's milk. They also included drugs such as cocaine, suggesting that their use by the residents of Tashkent was not unknown in the 1920s and supporting the evidence from other contemporary texts.

What kinds of results could the questionnaires used by Mol'kov's team and their associates yield? Based on their research instruments, social hygienists were able to draw a social portrait of *bytovoi *alcoholic, with a certain educational and professional background and with a certain monthly salary range. He or she would be of certain age, consume a given amount of alcohol on certain occasions and for different reasons (e.g. on religious holidays, at weddings or funerals, for the purposes of 'treatment' etc.) or without any reason at all, start drinking at a certain age, have his or her own drinking preferences, and spend a certain amount of money on alcohol per month. It would also be possible to make some statements about a drinker's living conditions and the quality of his or her family life and diet. Most importantly, one would be able to claim (or not to claim) the presence of all sorts of connections without having sufficient proof of the existence of causal relationships. As Solomon's research demonstrates, social hygiene researchers were often inclined to blunt the political thrust implicit in their work. For understandable reasons, they preferred to present a descriptive picture focused on several factors in the social milieu of the drinker "rather than on that milieu as a whole," backing away from "the trenchant criticism of Soviet society implicit in their analysis." Nevertheless, the picture that emerged from the research of social hygienists was grim and taking one step further would have revealed the need for major structural changes in the society [[10]: pp259-268].

With the changing political environment in the Soviet Union at the end of 1920s and with studies conducted by social hygienists on a variety of health and disease-related sensitive issues revealing a disturbing situation, the field of social hygiene came to an abrupt end by 1930. In January 1930, the field's chief patron, Public Health Commissar Semashko, was removed from his post and replaced by M. F. Vladimirsky, who had little experience in public health. The State Institute for Social Hygiene was transformed into a new organization dealing with administrative issues rather than public health research. The head of the Institute's alcohol research department and a fierce critic of the state's liquor policy, E. I. Deichman, was removed from the board of the Society for the Struggle against Alcoholism, whereas the All-Union Council of Anti-Alcohol Societies was formally abolished. It was announced that socialism and the socialist way of life themselves would gradually "destroy drunkenness." In September 1930, Stalin was giving directions to Molotov "to increase...the production of vodka" and "to aim openly and directly for the maximum increase in output" so that more funds could be raised for the military [11,24,30,31].

However, the decline in the authority of social hygiene research on drinking that began soon after the first findings were published, the demise of the field as well as the subsequent fall of *narkodispanser *cannot be fully explained without looking at the confrontation between 'hygienists' and the mainstream, clinical psychiatrists. To be sure, social hygienists' claims of hegemony over the treatment of alcoholics could not be successful without overcoming the opposition from a professional group, whose interests were directly affected. Without any expertise in treating chronic alcoholics and 'dipsomaniacs' that already put them in a disadvantaged position, social hygienists had to come up with an efficient policy and therapeutic approach to habitual drinking. However, their emphasis on "cultural work" and temperance propaganda appeared as a long-term strategy rather than a quick solution of the problem in a country which was undergoing rapid (and often forced) transformations by the end of the 1920s [[11]: p188]. Moreover, often run by 'social hygienists-narcologists', *narkodispansers *did not deliver as much as Sholomovich was promising. As a result, clinical psychiatrists easily emerged as winners, who by the end of the decade regained their monopoly over alcoholism and drug addictions.

Having suffered a fatal blow, the 'locomotives' of social narcology movement - Moscow-based *narkodispansers *- were stripped of their independence status and had to 'rejoin' their 'mother-psychiatry'. In 1932, they were no longer called '*narkodispansers' *and became part of neuro-psychiatric dispensaries [32]. As such, the disappearance of *narkodispansers *had little to do with the actual dynamics of drug use in the country and the regime's lack of interest in monitoring the drug situation.

Yet, this is not the end of the story, since by the time Moscow-based *narkodispansers *were merged with neuro-psychiatric dispensaries, the latter have been affected by the "great break" as well. Rozenshtein and his fellow mental hygiene advocates were accused of their intentions "to gain control of the entire public health" and of the 'overdispensarisation' (read 'overmedicalisation') of the 'healthy' citizens. With mental hygiene surveyors discovering mental or nervous illness in as many as 76 percent of teachers and 71.8 percent of medical workers, or, as was the case with the Tashkent-based psychiatric hospital, in 71.6 percent of nurses and nurse aids (*sanitary i srednii medpersonal*), the state's tolerance was exhausted. In 1931, the Russian government issued a resolution "On the Situation in Psychiatric Hospitals and the State of Affairs in Psychiatry in the Republic" prohibiting the opening of new institutes of preventive psychiatry in the capital city. Rozenshtein's institute was ordered to focus its scientific activity on the provision of psychiatric care in the republic, including such practical aspects as labour therapy. In 1932, Rozenshtein had to publicly denounce his own views and "to dissociate himself from everything alien" ("*otmezhevat'sia ot vsego chuzhdogo*"). He died two years later of natural causes, but his wife and his daughter were arrested by the NKVD (*Narodnyi komissariat vnytrennikh del*, People's Commissariat of Internal Affairs, the political and secret police of the Soviet Union) in 1937. As the mental hygiene movement declined by the early 1930s, neuro-psychiatric dispensaries' functions were reduced to "collecting medical statistics, provision of out-patient care, registration of patients, and referring them to psychiatric hospitals" [12,15,33-36]. They were now serving as a tool of the mainstream, clinical psychiatry, which considered habitual alcoholism and drug use as distinct mental diseases located within an individual, not within society.

### Opiate addiction in the hands of clinical psychiatrists

At the time when Sholomovich was conceiving the practical and theoretical bases of narcology and the ways it would fight against 'narkotism' independently from psychiatry, many other psychiatrists openly expressed opposing ideas and remained determined to pursue a different approach to the drugs issue. Thus, even before the first volume of *Issues of Narcology *appeared, L. Prozorov presented his version of the "struggle against narkotism" in the official publication of Narkomzdrav, which stressed the need for compulsory treatment of habitual narkomans (*privychnye narkomany*) and for the establishment of psychiatrist-managed therapeutic colonies for 'cocainists' [37].

A year later, in 1925, one of the most widely cited works on the 'constitution' of narkomans and the role of endogenous factors in 'genuine' drug addiction (*'genyinnaia' narkomania*) appeared in Moscow. It was published by a well-known Moscow psychiatrist, Mark Sereisky, who received his medical education in Munich in the early 1910s and worked for some time in Emil Kraepelin's clinic before returning to Russia. Relying on data collected in the psychiatric clinic of the First Moscow State University (which was a bastion of clinical psychiatry directed by a highly influential Professor of psychiatry, Petr Gannushkin), Sereisky argued that some people were initially predisposed to drug abuse. This was particularly relevant in regard to morphine addiction, where such an important social factor as access to morphine played a secondary role. "All physicians have the opportunity to obtain morphia," - he wrote, "yet, not all physicians are morphinists" [[38]: p24]. To support this basic observation with some clinical evidence, he studied the constitution of narkomans, 76 percent of whom consumed morphine, by looking at their character and heredity. According to his findings, as many as 96 percent of all patients had some 'aggravating factors' in their heredity. Furthermore, nearly 75 percent of cases which he studied had 'obvious pathological deviations' in their psyche, pre-morbid or "pre-narcotic" personality. Yet, in Sereisky's view, this was clearly an underestimate since those physicians who completed some of the older medical records did not pay as much attention to the personality as hid did. Thus, with his psychiatric gaze, Sereisky concluded that the first dose of morphine only served to 'close the circuit' and resulted in the development of the '*narkomannyi*' reflex, in line with Pavlovian teachings. Based on this concept, he took up the position of pre-revolutionary psychiatrists arguing that prognosis depended directly on heredity and suggesting that the treatment of narkomania usually led to a very disappointing outcome, making prevention a top priority [7,38].

As mainstream psychiatrists engaged in challenging social hygienists' bid for the right to manage habitual alcoholism, Sholomovich's elaborations on the need to differentiate between 'narkotism' and 'narkomania' became a target as well. In 1928, writing from a psychiatric clinic in Leningrad, Gorovoi-Shaltan argued that their 'material' (42 morphinists treated between 1919 and 1922) supported the conclusion that morphinism was caused by 'endogenous' factors. The next step that he made was to re-define 'morphinism' as "constitutional psychoneurosis complicated by chronic intoxication." While his definition was hardly ever accepted in its original form and the word 'constitutional' was immediately questioned by the editor, by drawing a connection with the earlier work of increasingly respectable and influential Mark Sereisky, he rather sought to reassert the psychiatrists' authority over the habitual drug user. Such a definition of morphinism, as he thought, also explained the well-established difficulty of treating it [[39]: pp 49-51].

Gorovoi-Shaltan, perhaps like every other psychiatrist who published on opiate addiction in Russia in the 1920s and who commonly reported a high percentage of 'medical addicts' in the sample of patients, also stressed that the most important avenue for prevention of morphine addiction was the medical profession itself and called for extreme caution when prescribing and administering morphine to patients. Apart from that, raising public awareness on narcotic drugs through 'enlightenment' activities and brochures was regarded as an appropriate prevention technique by many psychiatrists. Although it was something on which they agreed with hygienists, here the question of the content of messages to deliver became one of the most crucial ones. In the mid-1920s, when the social hygiene movement was at its heyday, the views of Sereisky and his like-minded colleagues were, as the book written by medical doctor E. B. Bluemenau clearly suggests, widely accepted and disseminated through a series of 'popular discussions on health and diseases'.

Published in Leningrad in the same year as Sereisky's paper, Bluemenau's book was concerned with the consequences and harm caused by the use of 'dope' (*durmany*) and provided a revealing description of drugs and drug users to larger audiences. In his general introduction to the issue of dope, Bluemenau asserted that drug users cannot be considered as "mentally healthy" whereas *durmany *were portrayed as powerful demons enslaving the ones who had tasted the forbidden fruit. Speaking specifically about opium and morphine, he 'promoted' them to the status of the "demons of humankind." Sounding like a replay of Sereisky, he wrote that "of course, it would be sad if all those who have ever been administered morphine, would become morphinists..." Yet, luckily, "[n]ot every person becomes a morphinist," he emphasised, since in order for that to happen, an individual needs to have a certain predisposition, whereas if a "normal person" gets exposed to morphia for some medical reason, this only results in some unpleasant feelings such as nausea and drowsiness, but they "all go away" after some time and "[t]his is the end of the business" [[40]: pp 40-51]. Reflecting the position of mainstream psychiatry, Bluemenau concluded his 'popular discussion' on the opiate dope (*odurmanivanie opiem i morfiem*) by stating that every morphinist was a "mentally abnormal person" who required "a long-term, skilful treatment in specialised institutions" [[40] p 61] .

However, even though mainstream psychiatrists believed that the treatment of opiate addiction was both an uneasy and an unpleasant task, there were still numerous questions central to the management of narkomania which needed to be addressed. Where and how to treat narkomania? Should a drug addict be treated on a compulsory basis? Given that he was mentally ill, could a Soviet drug addict enjoy the same rights as other, 'normal' citizens? What ought to be done with a female addict, especially if she was pregnant and the fetus reacted to abstinence like an 'accomplished' "opioman"? Should opiates be withdrawn from the patient in order to achieve the goal of complete abstinence? Or, perhaps, a scenario of 'the opium of the people' could still be applicable in Soviet society, where Marxist ideology prevailed and where by 1934, according to Aleksandr Matveevich Rapoport, the editor of the *Problems of Narcology *and the author of an editorial "On the Main Tasks of Soviet Narcology," "the digestion and re-working" of narcology's scientific heritage was impossible without taking "a strictly Marxist approach"? [41]

As discussed above, the conceptualization of opiate addiction treatment in early Soviet Union was profoundly connected with broader debates about the management of narkomania and narkotism, which also included chronic alcoholism and habitual drinking. Already in 1921, Kutanin was suggesting that all narkomans should be treated on a compulsory basis on the grounds that their condition was extremely difficult to manage, with voluntary treatment being highly problematic. Most importantly, they were also mentally ill patients who were dangerous to society [16]. Representing the official view of Narkomzdrav (which, nevertheless, was published as a discussion paper inviting the local authorities and individual comrades to send their feedback on the issue of the struggle against narkotism) three years later, Prozorov proposed to establish special units in large cities, which would function as registration facilities and as filters for the separation of 'occasional' narkomans from 'habitual' narkomans so that latter could be subjected to compulsory treatment. Although his rationale for compulsory treatment was primarily derived from examples related to 'cocainists', it also covered other drug addictions as well as grave cases of alcoholism. Cocaine addicts, he argued, were "socially-dangerous elements" committing anti-social acts and spreading the 'infection' around themselves. Since 'cocainism' in its essence was an 'infectious disease', Prozorov stated that the response to it should be commensurate with what is usually done by epidemiologists to prevent to an outbreak of any infection: one would need to "locate the source of infection"; to isolate the cases and to disinfect the carriers of infection; and to take care of "soil sanitation" [[37]: pp23-25]. When applied to drug addiction, this approach effectively called for identification and registration of narkomans, long-term isolation form society in compulsory treatment colonies, and a merciless fight against drug dealers.

Although no specific piece of legislation or inter-institutional instruction on compulsory treatment of narkomans seems to have been adopted in early Soviet decades,^g ^as early as 1927 the Soviet government issued two other instructions that established specific rules and procedures for compulsory treatment of a) alcoholics who constitute a social danger ("*Instruktsiia po Primeneniiu Prinuditel'nogo Lecheniia Alkogolikov, Predstavliaiuschikh Sotsial'nuiu Opasnost'*") [23]; and b) people with mental illnesses [10]. Passed by the joint resolution of the Narkomzdrav, NKVD and the People Commissariat of Justice in fulfilment of an earlier decree of Sovnarkom, the former was modelled on the latter and there is little doubt that both instructions well served the purpose of forced institutionalisation of narkomans, who (according to the formulations of the former instruction) would "constitute a social danger on the grounds of abuse" of narcotic drugs, "require special isolation" and yet were unwilling to submit themselves to "appropriate treatment" on a voluntary basis [23].

For Soviet psychiatrists, who perceived narcology as a sub-speciality concerned with questions related to narcotic drug and alcohol use, any discussion of the organization of narcological services in the country would normally address both addicts and alcoholics. It was not a coincidence therefore that when the first major textbook on the treatment of drug addictions appeared in the Soviet Union in 1940, many treatment institutions were described as appropriate for the management of both groups. Of great importance was the fact that the legitimacy of compulsory treatment for alcoholics *as well as for *narkomans, who were recalcitrant and socially dangerous because of their 'narkomania', was explicitly stated in that textbook. However, even more important was the emphasis on the use of labour as a form of therapy, since it was the labour colony which was deemed the most suitable institution for the long-term forced 'treatment' of narkomans and alcoholics and for making them accustomed to 'socially productive life'. The term of such 'therapeutic confinement' would normally range between three months and one year and, as the author of the textbook, Ivan Strelchuk, suggested, "if the whole affair is arranged properly, such a labour colony can exist on a self-supporting basis, without placing a burden on the state budget" [[42]: pp207-208].

Strelchuk, to be sure, was neither the first nor the last Soviet author to insist on the use of labour as part of the treatment, 're-education' and 're-socialization' of the morally depraved and anti-social alcoholics and narkomans. Although in a psychiatric asylum, for example, labour has long been regarded as an important component of the therapeutic regime, the emphasis on labour became much stronger for Soviet patients with drug addictions. As Prozorov claimed in 1924, one of the key differences in the treatment of addicts and other psychiatric patients was the extent to which labour can be applied in the therapeutic process. Thus, for the former labour had to be considered as fundamental, whereas for the latter it was only a necessary condition since their capacity for work was rather limited [37]. Even when deciding on cases eligible for treatment, Bakhtiiarov suggested that young morphinists who were 'useful' in terms of their productivity had to be given a priority. In Tashkent, doctors in charge of the in-patient drug treatment suggested that the 're-education' of addicts was part of the so-called 'active therapy'. With psychiatrists presenting narkomania predominantly as a disease to which a person was predisposed due to some kind of psychiatric pathology, the moralistic views concerning the treatment of opiate addiction gained a strong foothold in the psychiatric and narcological discourse in the late 1920s and 1930s. One author, calling for the renewed struggle against narkomania in 1931, went as far as to declare that, by far and large, morphinists were abject beings, who had a 'moral disability', lacked will and were totally useless 'elements' - both for the country and society [43-45].

One of the paradoxes was that these types of developments happened despite the fact that drug treatment specialists often had to deal with a 'medical addict' in their clinic, who was rather a 'victim' of doctors' careless prescriptions of opiates or a medical worker himself, and not with an anti-social, dangerous narkoman as he was portrayed in the early Soviet psychiatric literature. Thus, the majority of papers on the subject emphasized the role of biomedical connection in 'spreading narkomania' in various ways. In one of the earliest works written in 1921, Kutanin estimated that as much as 20 percent of his drug addicted patients were medical workers, with many of the rest 'commencing' their 'intoxications' following an injudicious advice of physicians and nurses [16]. In 1924, Prozorov considered morphinism, in a rather generalized fashion, a "professional disease of medical staff" [37]. Despite certain differences in their reported percentages of people who became addicted through the biomedical route, many other authors placed a strong emphasis on the significance of medical addiction, with considerable numbers of patients either initiating opiate use between the mid-1910s and the mid-1920s after receiving morphine injections from biomedical professionals or having a medical background themselves.

However, lest we rush into conclusion that the early Soviet drug addiction was a minor and isolated 'medical' issue among the aging cohort of opiate users, it would be appropriated to mention that some Russian historians had argued that in the first decade following the Bolshevik revolution the use of narcotic drugs was more prevalent in the Soviet Union than alcohol use. While any author making such argument would need to support it with substantial evidence that goes beyond what medical literature could possibly reflect and that covers an area far broader than the two major Russian cities of Moscow and Leningrad (with such evidence missing in the works of Russian drug historians), the information contained in contemporary medical literature suggests the presence of substantial drug use that remained beyond the reach of the doctor's office. For example, as Tashkent-based physician Kondratchenko confirmed, for as much as 60 percent of all patients treated in Tashkent in the late 1920s, seeking drug treatment was directly related to their inability to find enough money to procure increasingly expensive opiates. Often, these patients would come to the dispensary wearing torn boots because they "have spent everything on *shira*" ("*tak kak vse zagnal na shiru*") - an opiate well known locally. "But what if he had some means for opium?" - Kondratchenko asked a rhetorical question, and himself argued that "the necessity for seeking medical assistance in itself would disappear" [[28]: p1344]. Observations of Tashkent-based physicians were comparable to what Russian psychiatrists had seen in Leningrad in the 1920s and early 1930s. Among Golant's 189 patients, some of whom reportedly came from "all over the [Soviet] Union," only 15 asked for drug treatment after realizing the "destructive power of morphia." Others were driven by the need, as they could not afford to procure opiates on the black market, at clandestine dens. "Until the patient remains employed," - she wrote, "he almost never seeks for the treatment of morphinism" [[27]: pp17-29]. Furthermore, as early as in 1921, Kutanin argued that drug addicts hardly ended up in the 'catchment area' of specialized psychiatric institutions and that they asked for medical care only in the most severe cases. The question of where to look for these people seemed highly complicated until he accidentally found out that the large proportion of drug addicts were institutionalized in the penitentiary system [16]. On another occasion, writing from Leningrad in 1928, Gorovoi-Shaltan outlined diverse contexts of morphine use among people without contact with drug treatment institutions. Some of these accounts were seemingly told years ago by the patients, others looked more like anecdotal stories, and in one case Gorovoi-Shaltan referred to his own observations while working at the accident and emergency department. Yet, all of them offered some sense of what might have been taking place on the drug scene in which doctors were not present, at least not in the role of drug treatment providers:

*An officer from the front, with symptoms of traumatic neurosis, is hospitalized to lazaret, where four out of six female nurses are morfinistki. Among fellow patients - two are experienced morphinists and one is a beginner. In view of the [patient's] complaints about non-acute headaches and not quite good sleep, nurses, without knowledge of physician, offer morphine to the patient, while other fellow patients advice that he accepts the offer and provide a syringe for this purpose. The same officer, who gave up morphinization after one year, is mobilised again [and] ends up in a regiment, [where] he meets an old fellow-morphinist, and once again fails to resist the temptation*.

*Morphine is offered to the Party worker in a Soviet by his comrade, as a remedy for overcoming fatigue and for reducing a strain on the nerves, which had resulted from work[load] during Iudenich's advance on the city of Petrograd*.

*In a ration detachment, heading down to Ural for provisions, nine [servicemen] turn out to be morphinists, who then seduce the tenth [into morphine use], and on their way back this tenth [person] is already willing to give away everything in order to obtain morphine*.

*While working in the accident and emergency department...I had an opportunity to observe morphinism among prostitutes and other frequenters and visitors of various cafés and similar establishments. There, honestly speaking, morphine [use] was more often combined with other drugs [use], especially alcohol and cocaine *[[39]: p47].

Unlike medical workers, for whom opiate addiction was a 'professional disease' of exposure to morphine and syringes, militiamen and *chekists *(and people with apparent connection to the organs of power) relied on their 'authoritative profession' to support an already existing addiction and rarely, if ever, ended up as drug patients [45]. Yet, there was one other important group of opiate users that doctors were allowed to include neither in their formal records nor in medical publications, and these were Politburo members and some of the top echelon government workers. According to archival records from the Secret Department of the Communist Party Central Committee, Politburo members had very easy access to opiates in the late 1920s (and, to be sure, not only during that decade). Seemingly, they could also get drugs directly from Kremlin 'medical aid' kits, without any prescriptions and other forms of 'participation of the doctor'. Not surprisingly, some cases of habituation to drugs among high-ranking Soviet officials were not unknown, although never publicly admitted.

The other paradox was related to limiting the rights of drug users and prohibiting them from working. On the one hand, psychiatrists repeatedly stressed that labour was a vital part of the re-socialization focused therapy. Yet, at the same time they were among the strongest advocates for the major restrictions to be imposed on drug users' freedom to choose from the range of fields in which they can work or function when not in the specialised treatment/labour institution. It was not only and not mainly about the Soviet doctor addict who was to be deprived of the right to prescribe and practice medicine until he can prove that he or she is firmly and completely 'cured'. The nurse addict was in a significantly disadvantaged position because she or he was less indispensable to the Soviet biomedicine project, and, as Gorovoi-Shaltan proposed in 1928, if found to be addicted to morphine, nurses were to be 'removed' from the staff membership of their respective treatment institutions for good. While for the above two groups the issue of access to opiates was one of the factors that was considered by pro-restrictionists, access was clearly not the main driving force behind their calls for discrimination of drug users. Because addicts were viewed as 'morally depraved' and 'lacking will', Gorovoi-Shaltan argued that morphinists could not occupy any 'responsible' positions. Similarly, Bakhtiiarov argued that morphinists should not be allowed to operate a train or to work in the train station because this would be 'dangerous'. Since other authors have claimed that morphinists could supposedly 'infect' others with their pathological addiction, it seemed obvious for Gorovoi-Shaltan that they should be removed from the army and navy ranks and prohibited from residing in dormitories, where large numbers of people would be potentially at risk of contracting morphinism [39,43].

In the late 1920s and early 1930s the question of labour and the working class became so central to the Soviet government and its policies that it actually gave rise to the adoption of a new conceptual approach to the management of opiate addiction. Inspired by the works of Ernst Joël (1893-1929), which were published in Germany in the 1920s and first translated into Russian in 1930, this approach was based on the idea of maintaining morphine and heroin addicts on opiates in order to improve their social functioning and to promote the employment of drug addicts. As this will be discussed below, the maintenance of opiate addicts was not new in the Soviet Union at that time. However, the programme that was first introduced in Leningrad around 1930 by N. V. Kantorovich and his colleagues differed from others in its unique formulation. Kantorovich appeared to entirely share the views of Joël and agreed with him that the consumption of opiates per se did not lead to the loss of addict's capacity to work. According to Joël, the reasons for the addict's unemployment and suffering were rather constant concerns about obtaining morphine, the efforts required to secure a dose, fear, humiliation, financial problems and the necessity to lead an underground life. Based on this understanding, Kantorovich and his team decided to launch a maintenance programme (in fact, it was referred to as "provision of drugs" ("*snabzhenie narkotikami*") rather than maintenance, which reflected the Soviet experience of economic hardships necessitating the introduction of the policy of 'food provisions' and 'rations') for opium, morphine and heroin addicts and developed explicit rules and eligibility criteria for participant selection [Additional file 2]. The programme particularly targeted those "incurable" patients with chronic addiction who could potentially become both productive in terms of their ability to work and socially useful members of society. Anti-social, 'criminal addicts' who hit the rock bottom and underwent degradation were ineligible, as were cases without prior history of treatment and those who were treated 'insufficiently'. Technical aspects of the programme included the regulation of the dose and dispensing the habitual drug of choice (heroin, morphine or opium tincture) in the amount that would be sufficient only to avoid the appearance of withdrawal syndrome [46].

Guided by these rules and selection criteria, Kantorovich and his team were able to recruit 85 persons in their trial study over the course of six years. When in 1936 he first published the results of the study, he began with the challenging the long-established thesis of Sereisky on the role of exogenous factors in the aetiology of morphinism and suggested that any person, who systematically consumed morphia on a long-term basis, could develop a "deep habituation" to the drug. While he believed that the presence of psychopathological constitution facilitated this process, Kantorovich also claimed that it was not a necessary precondition. To support his argument he presented the findings from his study and illustrated them with the case histories of study participants.

According to Kantorovich, "good" results were achieved with regard to 40.1 percent of patients who received the so-called drug provision. Patient Sh. was one of the good examples. He used morphine and heroin during 18 years and was unsuccessfully treated at psychiatric facilities three times. Sh. was unemployed and had no place to live in. His whole body was covered with abscesses and phlegmons. Several days after Sh. was enrolled in the maintenance programme in late 1932, he found a temporary job. He was able to cope with that job and bought himself some clothes. He then found a permanent job of a doorkeeper at a dormitory. Sh. does his job very well and the management awarded him with bonus payments twice. Sh. stopped using drugs intravenously and switched to oral solution of heroin. He dresses very neatly, looks "ten years younger." He now keeps in touch with his ex-wife and wants her to come back.

Another 31.9 percent of patients achieved "satisfactory" results. Among these patients was female patient E. who had a twelve year-history of injecting morphine. She was enrolled in 1930 and since then remained in the programme. Throughout all six years of her participation in the maintenance programme, she was able to retain her job and provided support to her elderly parents. Patient E. occasionally spent money on illegal drugs and did not show up at work. This only happened when E. felt that she was unable to stay within the limits of prescribed doses. Finally, Kantorovich reported "the lack of positive results" in as little as 28 percent of all patients [[46]: pp73-74].

Nevertheless, against the backdrop of very promising results of a single study conducted by Kantorovich, on the one hand, and the far reaching claims of an outspoken group of Soviet psychiatrists calling for the use of labour therapy as a key instrument for enforcing a 'productive' discipline upon the unruly 'psychopath addicts' on the other hand, the overwhelming majority of the early Soviet drug treatment experiences in the first two decades of the Soviet rule were substantially different. The number of treatment colonies for drug addicts and alcoholics was very small, with alcoholics occupying most of the places. The remaining narcological facilities, including those that functioned within psychiatric hospitals, had a limited bed capacity and usually lacked workshops and any other facilities and conditions needed to organize labour therapy for their clients. As one opiate addict treated in Tashkent between 1929 and 1930 noted, "[I] wish I was able to occupy myself with something...[I am] lying [here in *narkostatsionar*] and have such a feeling as if the morphine prescription has been scratched by the nail inside my brain" [[44]: p88]. Even when such facilities were available to some extent in the existing narcological in-patient units, psychiatrists and narcologists soon realized that opiate addicts generally felt very weak after going through the process of withdrawal from drugs and were unable to do the kinds of jobs alcoholics would do. Moreover, outside of Russia, a significant number of cities and regions simply did not have any specialised drug treatment facilities at all.

Poor institutional and treatment capacities were in fact some of the main reasons why in many places in the 1920s and early 1930s local authorities were often willing to prescribe opiates to addicts and to have the issue addressed this way. Sometimes, dispensaries were willing to prescribe opiates to their clients during the period when there would be no bed places available, and would discontinue prescriptions at the time when the patient could be offered the possibility of undergoing an in-patient abstinence-based treatment. A certain number of state doctors were also running their private practices and in cities such as Leningrad, Voronezh, Odessa, Tashkent, Baku and Orel they played a key role in supplying drug users with opiate prescriptions [27,43,45]. However, outside of the large cities (most notably, Moscow and Leningrad, which many post-Soviet drug historians seem to consider as well representative of the history of narcotic drugs in the entire Soviet Union) there were major regional differences as well.

In the Russian Far East, for example, where no specialised drug treatment facilities were available, opium addicts of Amur Gubernia were provided with narcotic drug prescriptions by the local public health authorities from 1923. In return, narkomans had to commit to consuming drugs only inside their houses ("*v domashnei obstanovke*") and were punished with fines or compulsory work if found to take drugs in public. At the same time, in Vladivostok, the local administration decided to go in a completely different direction and placed all beggars, tramps and narkomans in a special 'house', which, however, had to be shut down after several months in view of the lack of funds to maintain this isolation facility. In 1924, beggars and addicts were forcibly transferred to the concentration colony on the Russian Island only to be abandoned in one year, when all available funding would run out [47].

In Turkmenistan, the demand for drug treatment was so great and the availability of specialised treatment institutions so limited, that in the late 1930s local authorities and psychiatrists had to think of establishing temporary dome-shaped felt tents (*yurts*) with each having five to ten beds in order to provide some sort of treatment to opium addicts. Such facilities had to be staffed at a minimal level, with one doctor and one nurse providing all the care after they would have received some basic instructions from a psychiatrist. Because of the very high prevalence of opium use in this country, deploying the psychiatric discourse and labelling local patients as 'psychopaths' would not seem possible for political reasons even by the late 1930s. Instead, writing in 1939 from the psychiatric clinic of the Turkmen Medical Institute, its director Professor E. V. Maslov had to describe opiate use in Turkmenistan using the language of social hygienists. There, according to Maslov, many opiate users first began to use the drug for therapeutic purposes, as a remedy against all diseases; a number of them became addicts-opium smokers because of the 'lack of culture' ("*nekul'turnost'*"); others learned how to use opium from older, experienced addicts. For such a 'backward' and 'uncultured' milieu, Maslov essentially had no other choice but to propose 'cultural enlightenment' - based prevention measures: brochures, posters, lectures and movies should all be aimed at struggling against opium [48]. Only a minority group of Russian injecting opiate users could be referred to as 'psychopaths' in Turkmenistan, and because their addiction was perceived as incurable they were able to receive morphine prescriptions at least until the mid-1930s [49].

In Tajikistan, no psychiatric hospital was operational until 1941, whereas the first specialized narcological office (*kabinet*) was opened only in the late 1950s. Although the first ten psychiatric beds were established in the infectious diseases hospital (!) sometime around the mid-1930s in the capital city of Tajikistan, they did not serve the narcological purposes and were designated for the temporary sedation and restraint of patients with acute psychiatric conditions. In view of the above situation, there was a common belief among Soviet Tajik psychiatrists and narcologists that until the 1940s, Tajik drug addicts were sent for specialized treatment to other psychiatric facilities located outside of the country, particularly to Uzbekistan. This suggestion was replicated in numerous publications and was particularly encouraged since it helped to sustain the myth of the benevolent Soviet state and its biomedicine project, which took care of the sick and was even willing to refer them for treatment to other republics [50-52]. The actual situation with regard to drug treatment in Tajikistan in the 1920s and 1930s was far more complex and differed dramatically from how it was described by Tajik psychiatrists. Tajikistan was allocated only two or three bed places in the Tashkent-based psychiatric/narcological institutions for people with mental illness and drug and alcohol addiction. Not surprisingly, when opiate users were occasionally sent to the Uzbek SSR, they happened to be non-native Europeans, not Tajiks. Among those few Russian people and other Europeans who were sent, many were insured, and in the late 1920s this became a very important factor in terms of receiving a priority access to any kind of medical care. However, in the early 1930s, the Institute of Physical Methods of Treatment (or Republican Physiotherapeutic Institute) was established in the northern part of Tajikistan, and as archival records from Dushanbe show, a small number of Russian morphine users who lived in Tajikistan at that time were referred to this facility as well. The types of therapies provided at this Institute included 'light therapy' ("*lechenie svetom*"), 'warmth therapy' ("*lechenie teplom*"), electric therapies, hydrotherapies, therapeutic mud-baths, massage and procaine block. When the staff of the Institute published their first scientific papers in 1940, it appeared that some diseases were also treated with "weakly radioactive waste of the local polymetallic industry" [53-60]. At the same time, in one geographically isolated region of the country, which had an extremely high prevalence of opium consumption among the local population and no drug treatment facilities in place, the 'struggle against narkomania' was conducted on the administrative 'front' and ended with the repression of opium users in the late 1930s.

In Central Asia, different types of opiates were used in different settings and situations by different groups. Yet, for various reasons, non-native European drug users were the ones who were most often in contact with drug treatment institutions in Uzbekistan during the early decades of Soviet rule. In 1934, without providing any specific figures, Leonid Antsyferov referred to the data of the Republican Psychiatric Hospital in Tashkent and claimed that the majority of 'hashishists', who "ended up receiving treatment," were represented by non-native, Russian people [[61]: p12]. Yet, none of the early Soviet Central Asian authors seemed to be willing to accept drug addiction as a 'European malady.' Often ready to consider their patients as representative of the Central Asian drug using populations and to generalize their conclusions, they refused to admit that the local population could possibly be "less infected" with drugs and preferred to locate the 'drug problem' within the 'natives.' As Kondratchenko wrote four years earlier, "the idea of treatment of narkotism" was just "not popular enough" among Uzbeks and warned that the small percentage of Uzbeks in his sample of outpatients was misleading [[28]: p1340]. Nevertheless, the facts that the Russian people represented the majority of 'narkomans' in contact with the Tashkent-based drug treatment institutions and that a great deal of early Soviet medical literature on drug addiction in Central Asia was effectively informed by the profiles of Russian and other non-native patients have never been acknowledged by later commentators on drug addiction in Central Asia. Instead, they commonly portrayed early Soviet drug use as part of the Central Asian custom and daily life (*bytovoe potreblenie narkotikov*) and reinforced the stereotype of an utterly 'traditional' drug use in Central Asia. In their accounts, Central Asian 'narkomania' was usually constructed as a disease resulting from indiscriminate administration of opiates by indigenous 'traditional' medical practitioners, the *tabibs*. The twinning of opiates and *tabibs *served the regime's political goal of exposing *tabibs *as 'evil' and eliminating 'backward' and 'ignorant' medicinal practices in Central Asian everyday life.

However, the differences between Central Asian patients and non-patients were reflected not only in their ethnicities and greater access or trust to the Soviet doctor, but also in the patterns of opiate use that these two groups have had. As one could more likely end up as a 'patient' in a drug treatment institution by using a certain opiate in a certain way or combination and for a certain period of time, so were the preferences of 'European' drug users divergent from those of the 'natives.' The use of *kuknar *as a 'traditional' native intoxicant, for example, has been widely reported by numerous commentators in pre-Soviet and Soviet Central Asia [Figure 1]. Yet, we do not see a single local 'kuknarist' among the narcological patients as they appeared in the early Soviet texts. This was equally the case in both Tashkent, where Uzbeks represented around 10 percent in one sample and 25 percent in another, and in Ashkhabad, where unlike in Tashkent 'the natives' constituted as much as 87 percent of patients receiving drug treatment [28,44,49]. Contrary to the images of 'miserable' people, who could hardly get rid of their 'evil passion' and thereby 'secure a few more years of life for themselves', as they were portrayed by some pre-Soviet, Imperial artists, administrators, physicians and ethnographers alike, the consumption of *kuknar *did not result in any substantial demand for drug treatment among the local populations throughout Soviet Central Asia [6,62-66].

**Figure 1:**
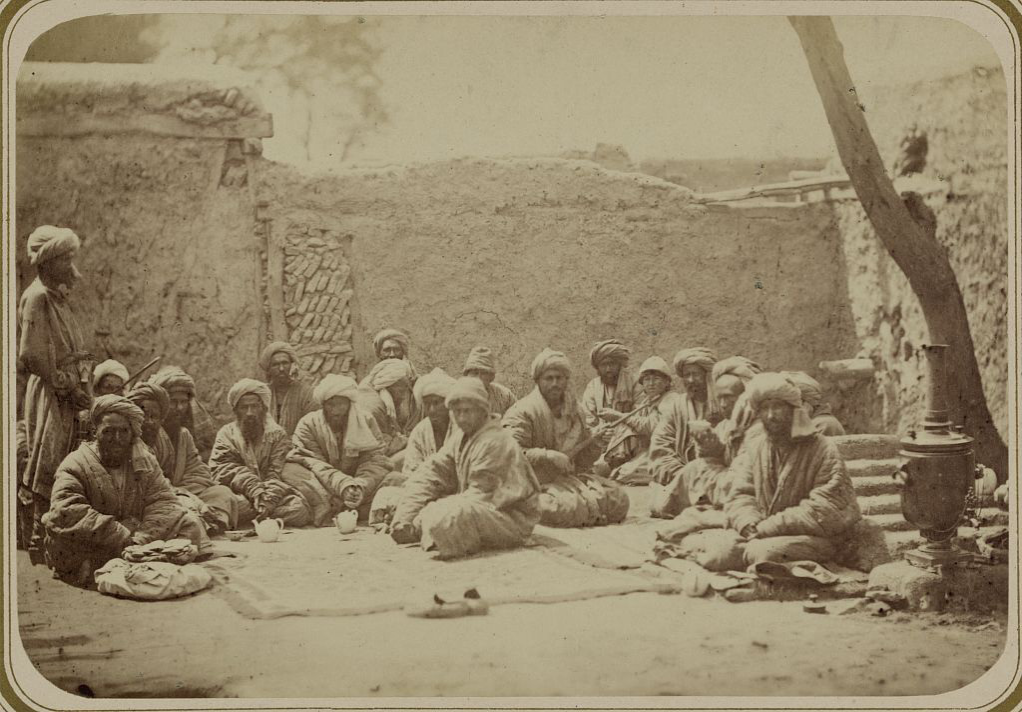
**Places of social gathering. Kuknarkhane. (Teahouse specializing in tea made from poppy pods). Turkestan Album. Ethnographic Part, 1871-1872**. Library of Gongress, Prints and Photographs Division, reproduction number LC-DIG-ppmsca-09953-00028 (digital file from Part 2, vol. 2, pl. 87, no. 289).

On the other hand, as Kondratchenko described in 1930, "only a declassed and decayed European opium addict, who is in a state of "*khumar*" [a local term for withdrawals commonly used throughout Central Asia], would consent to [consume] *kuknar*" [[28]: p1339]. Among the Russians, their tastes for purified opiates with high morphine content as well as polydrug use seem to have been important predictors of receiving a diagnosis of 'opiomania' and becoming a narcological patient. Thus, raw opium, *ter'iak *(also known as *tar'iok *and *tariak*), which the locals often used by dissolving it in tea, was avoided by the "Europeans," as suggested by Kondratchenko, because it had an "unattractive taste." Instead, they preferred to use *chakida *(Kondratchenko uses the term "*chigida*" and this is probably how the drug was known among the Russian-speaking users in the 1920s and 1930s) - a narcotic drug produced from raw opium by purifying and drying it through "a number of manipulations" and available in the form of very dark ("black"), thin plates. Another opiate preferred by the Russian patients, although surely used by the local people as well as Iranians and Afghans in early Soviet Uzbekistan, was called *shira*. According to Kondratchenko, *shira *was different from *chakida *in several ways: it had a lighter colour, higher content of morphine and a variety of mixtures in it, with hashish and *Strychnos nux vomica *sometimes added too [[28]: p1339]. In 1925, an Uzbek magazine *Er Uzi *published an article written by Boyish, who described the use of *shira *in the 'opium den' and claimed that it was common to add "*sukhta*" in *shira *while 'cooking' it, "so that *sukhta *would give its strength to *shira*" [[67]: p4]. *Sukhta *literally meant 'burnt' and was used as a term for residues of burnt opium, which would remain in the pipe for opium smoking. As for the morphine content, *sukhta *indeed was considered superior to *shira*. However, existing records indicate that *sukhta *was also used on its own, after it had been powdered, dissolved in water and boiled. It was sold in pre-loaded two-milligramme syringes at a price as cheap and as affordable as a bowl of *kuknar *and was reportedly injected hypodermically [28,49]. Although the presence of hypodermic and intravenous use of locally produced opiates on the Central Asian drug scene in the 1920s and 1930s may seem unexpected to many researchers concerned with drug use in post-Soviet Central Asia, the consumption of *sukhta *was reported as late as in the mid-1930s in Ashkhabad. It was injected both hypodermically and intravenously, and such usage was practiced exclusively by the Russian opiate users [49]. With cannabis, cocaine, heroin, morphine, dionine and codeine all available in early Soviet Central Asia, psychiatrists also decried polydrug use among the Russian patients and noted a nearly complete absence of this pattern of consumptions among the Uzbeks [28,44,68].

But, as far as the treatment of 'narkomania' was concerned, with the usual length of patient's stay in the drug treatment institutions in Russia and in other republics ranging between one to two months, and in some places like Ashkhabad narcological unit constituting on average 22 days, Soviet psychiatrists' appeals for the intensive use of labour therapy rarely materialized and often remained wishful thinking. Although in 1928 Gorovoi-Shaltan argued that the 'curing' of morphinism, rather than the withdrawal of morphine from the patient, represented the major challenge for the psychiatrist, the debates and discussions on the management and different methods of withdrawing opiates from patients in a clinical setting, in fact, became central to the development of narcology from the late 1920s and onwards [39]. As described in the medical literature on drug addiction published in the 1930s, these methods varied considerably throughout the Soviet Union. In Baku, the preference was given to the rapid withdrawal method. Samara-based psychiatrists introduced a gradual method in their facility, withdrawing opiates from their patients within a period of 30 to 45 days. Similarly, an abrupt method was usually avoided in Ashkhabad, unless patients themselves decided to go through the process of withdrawal without the help of therapeutic doses of either opium or morphine [45,49]. However, many leading institutions including the ones based in Moscow, Leningrad and Tashkent inclined towards withdrawing opiates from their patients abruptly.

One of the main reasons for this, as Golant explained in 1928, was to *reduce the number of days both patients and staff had to suffer*. In the Leningrad-based clinic where she worked, they initially withdrew opiates from their in-patients by gradually reducing the dose over the course of three to four weeks. While this was the case until 1925, the patient and his doctor were 'fighting' with each other every time the dose had to be reduced. Psychologically, this method appeared to be even more painful for the patient than an abrupt one, according to Golant, and it obviously caused serious inconvenience for the staff members who had to be able to obdurately refuse patients' pleas for the injection of an extra dose of morphine. The situation changed once the decision was made not to provide any opiates to the patients after they were admitted to the clinic. Although the patient's reaction to the abrupt withdrawal was quite 'stormy' ("*burnaia*"), Golant suggested that it normally lasted only for a very short period of three to six days and was followed by tangible improvements [27]. In Moscow, Strelchuk argued that the abrupt withdrawal method was the one that should be favoured for the sake of *saving resources and time*, which in practice implied that patients could be discharged very quickly. In Tashkent, doctors similarly asserted that they had no reasons to regret their choice of the abrupt withdrawal method and claimed that, with proper instructions given to personnel and with availability of necessary medications to address various symptoms that would occur after abrupt withdrawal, they were able to manage the addict very well [44,69]. The only problem, however, was that these kinds of optimistic views of the doctors were often not shared by their patients and, as the memoirs of a Russian-speaking "opium eater" from Tashkent on his "living through the symptoms of withdrawal" vividly explain, the actual experiences of the patients during the first week of an abrupt withdrawal were far more devastating than the word 'stormy' could possibly describe [70].

Patients' collapses and delirium, particularly among those whose health was considerably weakened by their opiate use and who probably constituted a substantial proportion of all cases, were some of the limitations of this method. Furthermore, it could not be used to wean pregnant women from opiates. Instead, as one paper published by A. Streliukhin in Turkmenistan in 1939 demonstrates, they had to be given morphine injections and withdrawn from the drug within one month after admission to the in-patient clinic if they were in the second half of their pregnancy period [71].

Serious health disturbances during the withdrawal periods, which were particularly pronounced in case of the abrupt method, also made doctors look for different ways of making this experience less dramatic to the patients without significantly increasing the bed space burden on chronically overcrowded and understaffed neuro-psychiatric and narcological institutions. *Calcium chloratum *was probably one of the most widely preferred substances that medical staff administered intravenously in order to warm up the patients and reduce pain. After western authors published their work proposing insulin and grape-sugar for the management of withdrawal syndrome, Strelchuk and other psychiatrists introduced the use of insulin to Soviet narcological practice as well. Oxygen therapy continued to be regarded as a 'very useful' addition to the repertoire of opiate addiction treatment specialists' for many decades after the initial experiments of Sholomovich and was praised in the third edition of Strelchuk's textbook published in 1956 [32,42,69]. However, one of the most fascinating and puzzling Soviet developments in the field of narcology was related to the invention of *gravidan *by Aleksey Zamkov (husband of a prominent Soviet sculptress, Vera Mukhina) in the late 1920s, and the use of this hormonal substance derived from the sterilized urine of pregnant women for the treatment of opiate addiction from the early 1930s up until December 1964, when the USSR Ministry of Health removed *gravidan *from its list of approved medicines.^h^

Despite Strelchuk's final admission in his 1940 textbook on the treatment of drug addiction that the rapid withdrawal method was "more humane" than the abrupt one, he still recommended the usage of the latter for all cases with good physical health and concluded that the abrupt withdrawal method had the largest number of supporters among drug treatment specialists. According to his textbook, the advantages of this method included the quickest removal of withdrawal symptoms which enabled the initiation of 'actual treatment of morphinism' as soon as three to four days after admission, with the minimal duration of patients' stay in treatment considered to be six months, - much longer than, perhaps, any early Soviet drug treatment institution was ever able to ensure! To what extent the 'actual treatment of morphinism' with bathing, shower, diet, three to four hours of labour therapy in fresh air, hypnoses, suggestion, and rational psychotherapy was performed in accordance with Strelchuk's advice in various in-patient facilities across the country that was on the brink of the war was another serious and problematic question, which had major implications for the Soviet psychiatrists' claims on the effectiveness of drug treatment [42].

## Conclusion

When in 1936 Kantorovich published his paper on the outcomes of a six-year trial of opiate maintenance treatment in Leningrad, this method appeared to be more effective in improving social functioning and employability as well as reducing criminality of the patients than it was ever reported by any other early Soviet author, who used either gradual, rapid or abrupt methods of withdrawing opiates from the addict. However, at that particular moment of the Soviet people's history, the state was about to launch its own 'effective' and the deadliest 'treatment' of 'criminals' and all other 'anti-Soviet elements' including drug addicts. The question of effectiveness of drug treatment was clearly not the most important one in the political and ideological environment of the Soviet Union in the late 1930s, and by suggesting that a small group of citizens should be given their 'opium' in a society that was aiming to become free from narkomania, Kantorovich was putting himself at enormous risk. The only way how he could possibly conclude in order to try to avoid arrest was to put in his last sentence the following text that we now can read in his article: "It goes without saying that the provision of chronics with narcotic drugs should be considered as a temporary, palliative measure, which is not anywhere near to ridding us of the need for further searches for radical therapeutic measures and of the repeated attempts to treat patients" [[46]: p74].

While unwilling to look back at their own professional past some post-Soviet psychiatrists and narcologists are still searching for the magic bullet against narkomania (and remain content with the Russian government's oppressive prohibition of opioid substitution therapy), Stalin's regime had found it already in 1937. In Moscow, where repressive measures were particularly focused on 'dens' (*pritony*) and their 'vicious inhabitants', the prevalence of drug use has decreased, according to one source, "only from the mid-1930s", down from 90 cases per 10 000 people in 1932 to 9 cases per 10 000 people in 1940 [72]. Although in 1940 the leading Soviet Moscow-based psychiatrist and drug treatment specialist, Ivan Strelchuk, was already celebrating "a nearly complete liquidation of narkomania in the USSR" in his textbook on the treatment of narkomania, and things seemed to be more or less sorted out both with the drug addicts and with the treatment of narkomania, the next major turning point occurred only one year later, when Germany invaded the Soviet Union.

Between 1941 and 1945, as one can easily imagine, the demand for legal opiates has skyrocketed in the Soviet Union. With wounded soldiers nearly dying in pain, opium became one of the state's top strategic commodities and all the harvest that was produced in the Kyrgyz SSR had to go 'to the front'. Anyone found to encroach on the opium harvest from the Kyrgyz poppy fields during WWII was shot on the spot as "the disorganizer of the rear and the enemy's ally" [[73]: p25]. The provision of the remaining chronic 'psychopath addicts', who survived the repression and did not end up in Gulag, with 'narcotic ration' would have been totally out of question. However, even before the war was over, the Soviet psychiatrist had to face an entirely different medical 'hero addict', and one who changed his approaches to drug treatment dramatically. As "two outstanding Soviet psychiatrists, who each have been in the field of addiction treatment...since the war," recalled in the late 1980s, "in the early post-war years, the larger part of narcotic patients [we had to deal with] consisted of morphinists who had become addicted in the course of extensive medical use of the drug as a painkiller during the war..." "They had nothing in common with those slimes you face nowadays...they were serious, positive men, soldiers. It was not their fault..." [[74]: p32].

The ways in which some of these 'positive', innocent men would later discover themselves in the shoes of the Russian WWI veterans and undergo a crucial transformation from 'medical addicts' to 'psychopath addicts' constitute an issue which is ripe for scholarly research. But back in the 1940s, the state had to first pay its tribute to the 'hero addict', who sacrificed everything during the Great Patriotic War and saved the Motherland. Placing him in a psychiatric asylum or a neuro-psychiatric *dispanser*, which lacked personnel, lacked medicines, lacked food and was catastrophically overcrowded, and making him go through the abrupt withdrawal method which he could hardly survive, would be an ultimate disgrace to the Soviet state [35].

In this situation, not only the maintenance treatment had to be resumed. Starting from 1948, Moscow psychiatrists began to use systematically a new method of 'demorphinisation' of the medical addict while he was in a deep, long-term *amytal-natrium*-induced sleep. According to Strelchuk, the introduction of this method was necessitated by the requirement to 'humanize' the treatment of morphinism. It relieved the patient of debilitating sufferings and prevented the occurrence of possible complications caused by the use of other methods of opiate withdrawal, including fatal and non-fatal cases of collapse and delirium. The price that the state was willing to pay for a humane demorphinisation of the 'hero addict' was incomparable to what was ever available to his fellow 'psychopath addict': round-the-clock duty nurse sitting at the hero's bedside for about two weeks and checking and recording, while he was asleep, his pulse, temperature, arterial tension and breathing on an hourly basis; feeding the patient and helping him into the toilet; and placing a hot-water bag near his limbs [32]. Similarly, in Tashkent, psychiatrists called for the broader use of nicotinic acid in order to alleviate the withdrawal symptoms and considered this approach to be particularly relevant to the treatment of the war time addicts, the so called 'hospital narkomans' [75].

Ironically, at the very same time public health officials were proudly declaring the entire absence of drug addiction in the Uzbek SSR (and elsewhere), which supposedly had disappeared due to the Soviet rule's concern for the culture, education and medical care of the Soviet people [76]. In fact, however, the Soviet cultural enlightenment project had little in common with specifically addressing drug use, and, as I have demonstrated in this review, the Communist authorities 'eliminated' addiction in the course of the administrative and repressive struggle against narkomania, - the struggle, in which the Soviet doctor failed and the NKVD officer 'succeeded' by murdering and incarcerating, not treating.

## List of abbreviations

The following abbreviations are used within the manuscript text: Narkomzdrav-*Narodnyi komissariat zdravookhraneniia*, People's Commissariat of Public Health; NKVD-*Narodnyi komissariat vnytrennikh del*, People's Commissariat of Internal Affairs; Sovnarkom-*Sovet narodnykh komissarov*, the Council of People's Commissar.

## Competing interests

The author declares that they have no competing interests.

## Endnotes

^a ^While many leading Russian psychiatrists were fluent in foreign languages, it seems that Erlenmeyer's (and Sollier's) work on the treatment of morphine addiction first became available in its Russian version only in 1899. Burkart's monograph on the chronic morphine poisoning (*khronicheskoe otravlenie morphiem*) was published in Russian in 1882 [42,46];

^b ^Many papers from the first volume of *Voprosy Narkologii *were initially presented and discussed at the First Scientific Conference on the Questions of Narkotizm that was organized in Moscow, in December 1923, following the All-Russian Conference on the Questions of Psychiatry and Neurology;

^c ^As presented by Sholomovich in different papers, these numbers of new patients contain discrepancies [18,25];

^d ^It is worth mentioning here that in post-Soviet Russia there seems to be a continued interest in the use of oxygen for the treatment of drug and alcohol dependence [77];

^e ^In 1931, for example, speaking primarily about neuro-psychiatric dispensaries, Prozorov wrote that the number of repeat visits was particularly low in those dispensaries, which did not have their own equipment for physiotherapy and could not refer their patients to such services (that is to say, in those dispensaries, which provided 'words of advice' and 'talkative therapies') [17];

^f ^The paper was first presented in January 1924 at the Second All-Russian Congress on Psychoneurology in Leningrad;

^g ^For example, in the early 1930s, Kamaev was one of the authors who continued to stress the need for the organization of compulsory treatment of narkomans, placing this under the rubric of 'the struggle against narkomania on the administrative-legislative front' [45];

^h ^There is a growing number of journal and newspaper articles being written on the history of *gravidan *therapy and its use in Soviet narcology [78-81], along with existing published primary sources [32,42,49,69]. In Central Asia, the use of *gravidan *was first reported 1936 by B. Smirnov, who was writing from the narcological unit of a psychiatric hospital in Ashkhabad, Turkmenistan. According to Smirnov, the use of *gravidan *did not make much difference on the patients' reaction to withdrawal of opiates. The feedback from the patients was often negative as they complained that *gravidan *did not help to alleviate *khumar *(withdrawals), with injections causing only more pain [49].

## Supplementary Material

Additional file 1 **Abstract. The Soviet doctor and the treatment of drug addiction: "A difficult and most ungracious task"**. A Russian translation of an abstract of this article. Click here for file

Additional file 2 **N. V. Kantorovich. Dispensary observations over morphine users**. An article on the results of opiate maintenance treatment study in Leningrad, USSR, 1930-1936. Click here for file
