# Supplementary material for: The Soviet doctor and the treatment of drug addiction: "A difficult and most ungracious task"
Source: Harm Reduct J. 2011 Dec 30;8:32. doi: 10.1186/1477-7517-8-32 (PMC3275499; doi:10.1186/1477-7517-8-32)
Supplement: Additional file 1 — Abstract. The Soviet doctor and the treatment of drug addiction: "A difficult and most ungracious task". A Russian translation of an abstract of this article. [file 1477-7517-8-32-S1.PDF]

Советский врач и лечение наркотической зависимости: «Трудная и в высшей степени неблагодарная задача»

Алишер Латыпов

Евразийская сеть снижения вреда,
информационно-исследовательская программа
г. Вильнюс, ул. Швитригайлос 11Б, Литовская Республика, 03228

Центр изучения глобального здоровья в Центральной Азии
при Колумбийском Университете,
1255 Амстердам авеню, Нью-Йорк, NY10027, США

E-mail: alisher@harm-reduction.org ; alisher_latypov@hotmail.com

Резюме

В данной статье содержится обзор развития подходов к лечению наркотической зависимости, применявшихся в Советском Союзе в раннем советском периоде. В центре внимания - институциональная борьба между ведущими специалистами социальной гигиены и психогигиены с одной стороны, и клиницистами-психиатрами с другой – что явилось решающим моментом для медицинской специальности, которая получила в Советском Союзе название «наркология». С этой отправной точки я перехожу к рассмотрению завоевывавших популярность и затем ушедших в забытье разных методов лечения, а также концептуализации понятия наркотической зависимости в научных центрах России, и отслеживаю, как это переносилось (или нет) в другие советские республики. Так как представители клинической психиатрии одержали неоспоримую победу в борьбе со специалистами социальной гигиены и психогигиены, весь арсенал наркологии был подчинен задачам основного направления в психиатрии. Хотя в чем заключалось это «основное направление» было не совсем ясно. Когда в 1934 году Александр Рапопорт настаивал на необходимости переработки системы наркологических знаний с точки зрения строго марксистского подхода, он смог только поставить вопросы и признать, что для их решения почти

отсутствуют «диалектически освещенные научные данные». Поддерживающее лечение потребителей опиатов, которое оказалось наиболее эффективным по результатам 6-летнего исследования, опубликованным в 1936 году, в конечном счете, было не созвучным с политической и идеологической атмосферой конца 1930-х годов. Такое «снабжение» пациентов наркотиками через диспансер рассматривалось как до некоторой степени временная мера в условиях, когда отсутствовали «радикальные лечебные мероприятия» для освобождения советского общества от наркомании. Когда Большой террор развернулся по всему Советскому Союзу, сталинский режим достиг своей цели по устранению наркотической зависимости с поверхности общественной жизни, загнав потребителей опиатов в глубокое подполье и отправив многих из них в тюрьмы и лагеря Гулага. В заключительном разделе я кратко анализирую изменения во взглядах на употребление наркотиков во время Второй мировой войны и описываю последующую реорганизацию действий советской власти в ответ на послевоенную ситуацию с опиатной зависимостью.

Ключевые слова: советская наркология; история; социальная гигиена и психогигиена; психиатрия; лечение зависимости; поддерживающая терапия опиатами; репрессии против потребителей наркотиков.
