# Supplementary material for: The Soviet doctor and the treatment of drug addiction: "A difficult and most ungracious task"
Source: Harm Reduct J. 2011 Dec 30;8:32. doi: 10.1186/1477-7517-8-32 (PMC3275499; doi:10.1186/1477-7517-8-32)
Supplement: Additional file 2 — N. V. Kantorovich. Dispensary observations over morphine users. An article on the results of opiate maintenance treatment study in Leningrad, USSR, 1930-1936. [file 1477-7517-8-32-S2.PDF]

Год издания XII *№ 3, 1936 года*

СОВЕТСКАЯ ПСИХОНЕВРОЛОГИЯ

ДИСПАНСЕРНЫЕ НАБЛЮДЕНИЯ НАД МОРФИНИСТАМИ[1]

Доц. Н. В. Канторович (Ленинград)

Из Наркологического отделения Василеостровского невропсихиатрического диспансера (главврач М. М. Блюмкина) и Невропсихиатрического института им. Бехтерева в Ленинграде (директор О. С. Фридман)

Говоря о морфинизме, в дальнейшем изложении мы будем иметь в виду также героинизм, опиофагию и привычку к употреблению других алкалоидов опия.

Излагаемые в дальнейшем данные и соображения основаны на изучении материала наркологического отделения Василеостровского невропсихиатрического диспансера, в котором централизовано обслуживание больных наркоманов (кроме алкоголиков) для всего Ленинграда и где в течение шести лет были прослежены нами 125 случаев и подвергнуты неоднократным обследованиям (на дому и на производстве) и проведена статистическая обработка материала.

ОБЩАЯ ХАРАКТЕРИСТИКА МАТЕРИАЛА

Среди наших больных было 65,6% мужчин и 34,4% женщин.

Интересно, что в прежнее время авторы указывали на резкое преобладание женщин (90%) среди морфинистов [Маршалл, Ирль, Гулл, Терри, Броун, Фар (Marschall, Earle, Hull, Terry, Brown, Farr)]. В новейшей же литературе указывается на противоположную тенденцию. Так Гамильтон (Hamilton) в 1919 г. нашел 90% мужчин и всего 10% женщин. Серейский указывает, что мужчин среди морфинистов в четыре раза больше, чем женщин. К аналогичным выводам пришла Голант; для выяснения причин колебаний полового состава морфинистов необходимо еще дальнейшее изучение этого вопроса.

Большинство наших пациентов заболели в возрасте от 20 до 30 лет, что совпадает с данными Голант. В отношении длительности заболевания мы имеем следующие цифры: до 3 лет больны 10%, до 10 лет 41%, до 20 лет 42%, свыше 20 лет 6%. По социальному составу преобладает группа служащих — 68,8%, рабочих всего 9,6%, остальные пенсионеры 9,6% и „прочие" (12%).

Среди наших больных 63% со средним образованием и 16% с высшим. Среди морфинистов представляет некоторые особенности в отношении социальной характеристики, лечения и прогноза группа соматических больных („Fortuitous" по Hubbard'у).

Сюда относятся лица, страдающие хроническими заболеваниями, сопровождающимися сильными болями или приступами, в связи с чем они в течение длительного времени принимали по назначению врачей морфий (бронхиальная астма, туберкулез кишечника, спинная сухотка, желчные камни и т. д.). В большинстве случаев у них дополнительно к основному заболеванию развивается морфинизм.

В отношении этих лиц, как правило, можно высказать безнадежный прогноз по поводу излечения морфинизма при неизлечимости основного заболевания, но сама наркомания у них проявляется обычно в более мягких формах. Таких „соматически больных" в нашем материале было 22%. При семейных наркоманиях чаще наблюдается морфинизм супругов, реже родителей и детей, братьев и сестер и т. д. Мы имеем в нашем материале 15% семейных наркоманий. Анализ материала показывает, что в этиологии семейных наркоманий имеет значение прозелитизм, соблазн, браки между морфинистами. Гораздо меньшую роль играет наследственно-конституциональное предрасположение. Морфинизм в известной степени является профессиональным

[1] Помещая настоящую статью, Редакция приглашает читателей высказаться по поводу рекомендуемого в ней метода лечения.

заболеванием медицинских работников. У нас медработники составляют 29,6% всех больных. Среди медработников 5,4% младшего персонала, 13,5% врачей и 81,1% среднего медперсонала. Такова, вкратце, общая характеристика нашего материала.

ЭТИОЛОГИЯ

Одним из кардинальных вопросов является этиология морфинизма. Чрезвычайно распространен взгляд на морфинизм как на проявление психопатии.

Так Спрейг (Sprague) различает маленькую группу психически полноценных (соматические больные), большую группу моральных дегенератов и самую большую группу дефективных и преступников. Вулей (Wholey) делит морфинистов на циклотимиков и конституционально - имморальных, Гендерсон утверждает, что большая часть морфинистов являются психопатами, а Блок идет еще дальше, заявляя: „Нормальная личность никогда не может сделаться морфинистом".

Далеко не все, однако, являются приверженцами такой конституциональной точки зрения.

Так Хуббарт, являющийся директором Нью - Йоркской городской наркологической клиники пишет: „Среди морфинистов есть конституционально малоценные и высокоценные индивидуумы, слабоумные и богато одаренные, морально низко стоящие и люди высоких моральных качеств". Умеренное значение придает конституциональным факторам и такой знаток вопроса как Иоэль (Ioël). Голант нашла только 22% несомненных психопатов среди мужчин морфинистов и 12% среди женщин.

Мы тщательно выясняли семейный анамнез и преморбидную личность наших больных, причем оказалось, что 48% из них были полноценными в невропсихическом отношении. Даже при большом желании не было никаких данных для отнесения их к категории психопатов. Остальные 52% больных, с бо́льшими или меньшими основаниями, нами отнесены к категории психопатов. Из соотношения между нормальными и психопатами, мы делаем вывод, что наличие психопатии предрасполагает к заболеванию морфинизмом, но отнюдь не является обязательным. Соматическая конституция морфинистов не дает каких - либо характерных особенностей, что подтверждает нашу точку зрения.

Что касается типа психопатии у морфинистов (имея в виду преморбидную личность), то мы получили следующие данные (в абсолютных цифрах): параноидов — 9, психастеников — 13, истериков и с различными извращениями — 20, неустойчивых и прочих психопатов — 13. Эта пестрая, в общем, картина указывает всетаки на преобладание психастеников, неустойчивых и истериков.

Значительный интерес представляют субъективные причины и поводы к развитию морфинизма. Мы распределили их на четыре группы. К первой относится привыкание в связи с медицинскими назначениями. Эта группа является наибольшей, к ней отходит 48% всех больных. Дальше идет „самолечение". Сюда мы относим случаи когда привыкание к морфию развилось на почве первоначального применения морфия самим больным с лечебной целью по поводу болей или тяжелого самочувствия, вызванного каким - либо неприятным переживанием. В третьей группе привыкание к морфию вызвано соблазном и примером других морфинистов, и последнюю группу образуют лица, начавшие употреблять морфий из любопытства и желания испытать новые ощущения. Во всех этих группах (за исключением группы медицинских назначений) мы имеем приблизительно одинаковое (по 16 - 17%) число больных. При рассмотрении субъективных причин отдельно у мужчин и женщин можно отметить преобладание „любопытных" среди мужчин (16% мужчин и только 1,6% женщин). Наоборот, пример и соблазн играет большую роль в отношении женщин. Интересно, что в группе „медназначения" сравнительно мало психопатических личностей, среди ставших же морфинистами из любопытства — психопатов 85%. Мы подробнее остановились на субъективных причинах морфинизма потому, что они дают нам некоторые указания относительно медицинского и социального прогноза. Так группа „медназначений" дает несомненно лучшие предсказания, чем „любопытные".

Резюмируем наш взгляд на этиологию морфинизма следующим образом: *глубокое привыкание к морфию может возникнуть у каждого человека подвер-*

гающегося в течение более или менее длительного времени систематическому воздействию наркотика. Наличие психопатической конституции, нервнопсихическая неустойчивость, вызванная экзогенными, социальными факторами (реактивные состояния), а также незакончившийся процесс формирования личности облегчают это привыкание, т. е. развитие морфинизма, но не являются обязательными условиями для развития болезни. То обстоятельство, что у некоторых лиц не развивается привыкание, даже после длительного введения им морфия, не опровергает нашей точки зрения.

Учитывая причины и поводы к развитию морфинизма, необходимо с целью профилактики:

1) усилить борьбу с нелегальной продажей наркотических веществ;

2) ограничить и регламентировать отпуск наркотиков из аптек, в частности изъять из свободной продажи, так называемые „иноземцевы капли“ (*опий и эфир*);

3) *фиксировать внимание врачебной общественности на последствиях неосмотрительного назначения* наркотических веществ больным (пример: длительное назначение героина при легочном туберкулезе в некоторых учреждениях);

4) предусмотреть в программах медвузов и медтехникумов обязательное ознакомление студентов с наркологическими сведениями.

ВОПРОСЫ ДИСПАНСЕРИЗАЦИИ МОРФИНИСТОВ

Ограничимся рассмотрением результатов стационарного лечения, которое большинство из наших больных проходило в различных психиатрических учреждениях, причем многие из них лечились неоднократно. Оказалось, что в 52,5 % больные возвращались к приему морфия в день выписки из больницы, в 36,1 % случаев рецидив наступал через различные, обычно очень короткие сроки и только в 11,4 % мы могли констатировать более стойкое излечение со сроком воздержания от наркотиков свыше трех лет после лечения. Интересно, что в группе выздоровевших преобладают нормальные женщины, наихудшие же результаты дают психопаты-мужчины. При существующих методах лечения морфинизма, только незначительная часть больных может быть излечена. Большинство же морфинистов приходится считать неизлечимыми хрониками. Прежде чем поставить вопрос о судьбе этих хроников, скажем несколько слов об их психологической характеристике.

Следует различать психопатические черты преморбидной личности морфинистов от тех изменений, которые вызываются хронической морфийной интоксикацией и создают своеобразный „морфийный“ характер. Невыполнение этого правила послужило источником ошибки для многих авторов, относивших всех морфинистов в категорию врожденных психопатов. Характеризуя личность морфиниста, надо иметь в виду, что она выявляется различно в зависимости от того, находится ли больной в состоянии эйфории, вызванной усиленной наркотизацией, в состоянии морфийного голодания или в состоянии известного временного равновесия, наступающего при употреблении привычных доз наркотика. Считая последнее состояние *обычным, нормальным для хронического морфиниста, мы и исходим из него при характеристике личности.*

Морфинисты робки, застенчивы, внушаемы, обидчивы или раздражительны, впечатлительны, лживы, доверчивы, легкомысленны, щедры, добры, не настойчивы, мягкие пессимисты. Они избирательно контактны. Наблюдается выраженный комплекс малоценности с гиперкомпенсацией. Сознавая себя больными, недостаточно приспособленными к жизни людьми, они вместе с тем считают себя стоящими в каком-то отношении выше, значительнее и ценнее других людей. Морфий как бы открывает им другой мир, недоступный прочим смертным. По правильному наблюдению Голант, морфинисты долго сохраняют хорошие взаимоотношения с семьей и родными, несмотря на материальный ущерб, который они им приносят. Антисоциальность морфинистов проявляется в специфических преступлениях, связанных с добыванием наркотика (подделка рецептов, мелкие растраты) и в меньшей степени в прозелитизме, соблазне и приучении других лиц.

Вопрос о трудоспособности морфинистов заслуживает особого и тщательного рассмотрения, тем более, что в специальной литературе он или вовсе не освещается или затрагивается вскользь. Так Голант среди мужчин морфинистов нашла 78 % лиц, не могущих работать вследствие морфинизма. Большинство из наших

больных в момент обращения в диспансер отличались значительно пониженной трудоспособностью, или же были нетрудоспособны.

Для суммарной оценки трудоспособности группы морфинистов могут служить следующие данные, вычисленные в отношении всех морфинистов, опиофагов и героинистов, прошедших через наш диспансер за шесть лет. Сюда входят, следовательно, лечившиеся и нелечившиеся, получавшие снабжение и не получавшие таковое. Для подсчета взяты последние сведения, имевшиеся в историях болезни.

Работают удовлетворительно и хорошо	43,2%
Работают плохо (прогулы, перемена места, частые обращения за отпуском по болезни)	30,4 %
Не могут работать вследствие наркомании	12,0 %
Прочие (пенсионеры, домохозяйки) и не выяснен.	14,4 %
Итого	100 %

Выполняемая работа соответствует квалификации	71,7 %
" " не соответствует (ниже) имеющейся квалификации	28,3 %
Итого	100 %

Чрезвычайно ценные указания для понимания причин нетрудоспособности при морфинизме дает Иоэль: „*Следует особенно подчеркнуть то обстоятельство*,— говорит Иоэль, *что морфинист уже вскоре после начала своей наркомании ничего не получает от своего наркотика, кроме устранения недомоганий, вызванных его отсутствием. Круг замыкается и попытка вырваться из него ведет к усилению мучительных явлений воздержания, которые в свою очередь вызывают повышение дозы. В этом периоде морфинист может, правда, сохранять работоспособность, если будет следить за своевременным снабжением своего организма морфием, но не потребление морфия, а именно постоянная забота, тщетные и унизительные попытки добыть морфий, страх, необходимость скрываться, денежные затруднения — вот что служит в это время источником многих страданий и понижения работоспособности этих больных*".

Всецело разделяя приведенные взгляды Иоэля, мы решили предпринять опыт снабжения неизлечимых хроников-морфинистов (также героинистов и опиофагов) наркотиками через диспансер. В настоящее время в нашем распоряжении имеются тщательно проверенные данные шестилетнего опыта проведения этого мероприятия, почему мы и считаем возможным опубликование их в печати.

Методика снабжения неизлечимых морфинистов сводится к следующему. Каждый вновь обратившийся в диспансер больной берется на учет и подвергается диспансерному наблюдению и изучению. При помощи аппарата социальной помощи, он обследуется на дому и на производстве, в диспансер вызываются родственники для получения необходимых сведений. Лишь после того, как больной достаточно изучен в психиатрическом и социальном отношении (на что уходит около месяца), решается вопрос о снабжении его наркотиками, на основе следующих правил:

1) морфинисты, ранее не лечившиеся, лечившиеся недостаточно и вообще случаи, где можно надеяться на успешный результат лечения, на снабжение не принимаются, а направляются в соответствующее учреждение для стационарного лечения. На снабжение принимаются только неизлечимые хроники;

2) глубоко деградированные психически и социально, криминальные и антисоциальные морфинисты снабжению не подлежат;

3) снабжению подлежат больные, которые могут быть трудоспособными и социально-полезными членами общества, при условии регулярного приема ими определенной дозы привычного наркотика;

4) больному выдается привычное для него наркотическое вещество (морфий, героин, настойка опия) в наименьшей дозе, при которой не возникает явлений абстиненции. Доза эта устанавливается путем специального изучения в каждом отдельном случае;

5) результаты снабжения проверяются систематическими повторными медицинскими и социальными обследованиями.

Техника снабжения хроников несложна. Диспансеризация наркоманов централизована в одном учреждении города. Больные получают от диспансера направление в Медснабтрест с предложением выдавать им в течение известного срока определенное наркотическое вещество в прописанной дозе. Обычно прикрепление выдается диспансером через Медснабтрест каждый раз сроком

на месяц. Из аптеки же больные получают не свыше трех или пятидневной дозы сразу по указанию врача. Прописываемые дозы индивидуально различны. Морфий, пантопон и героин прописываются в одно- или двухпроцентном растворе. Большинство больных получают 3—5 грамм двухпроцентного раствора pro die, а в отдельных случаях и больше, до 10 грамм. Принимаются меры к предотвращению возможной подделки рецептов (специальные бланки, все цифры указываются прописью). Вначале, некоторые больные не ограничиваются назначенной им дозой, расходуют ее раньше времени, являются в диспансер за дополнительным рецептом и т. д. Довольно быстро, однако, удается дисциплинировать их и привить сознание того, что всякое нарушение назначенной дозы влечет за собой неприятные последствия и что кроме прописанного количества больной ничего получить дополнительно не сможет. Никаких инъекций в диспансере или выдачи наркотиков непосредственно на руки больным мы не производили.

Приведем несколько кратких выдержек из историй болезни морфинистов, принятых на снабжение.

1. Больной Ш., 34 лет, фармацевт. В течение 18 лет принимает героин и морфий, доходил до 1,5 morhii mur. pro die. Два раза безрезультатно лечился стационарно. За последние три года совершенно опустился. Жена от него ушла, лишился квартиры. Все имущество больного заключается в комплекте крайне поношенной одежды. Не работает, живет в ночлежке. Нищенствует. Собранное подаяние тратит на наркотики. На теле флегмоны и абсцессы. Истощен. Предложено стационарное лечение, от которого больной отказался. Спустя несколько месяцев явился вновь. Температура 40.3. На теле несколько десятков флегмон и абсцессов. Весь покрыт перевязками. Распространяет тошнотворный запах гноя. Летом 1932 г. помещен во вторую психиатрическую больницу, где пробыл около четырех месяцев. Отмечались тяжелые явления абстиненции, опасность летального исхода. В дальнейшем прекрасно поправился физически, но чувствовал вялость, некоторую апатию. С момента лишения наркотиков развилось расстройство аккомодации, диплопия, с которой больной и выписался. По выписке пытался работать, но не мог из-за расстройства зрения и психической вялости. Через две-три недели после выписки вернулся к наркотикам и к прежнему плачевному положению. В конце 1932 г. принят на снабжение. Через несколько дней поступил на временную работу. С работой справлялся, обзавелся необходимым бельем и вещами. Поступил комендантом в большое общежитие. Хорошо справляется с работой. Дважды премирован Работает свыше годе. Прогулов не было, отпусками по болезни не пользовался. Строго ограничивается прописанной дозой (5,0—2% героина в день). От внутривенных инъекций отказался. Флегмон и абсцессов нет. Хорошо одет, внешне стал неузнаваем „помолодел на 10 лет", по отзывам окружающих. Ведет переговоры с женой о ее возвращении.

2. Больной В-р явился в диспансер в 1930 г. в пальто, которое одолжил у родственника. Все имущество больного состояло из одной пары белья. После ранения на фронте в связи с медицинскими назначениями привык к подкожному введению морфия. Стационарное лечение не дало результатов. Имеет родных, живущих в хороших материальных условиях, но последние ничем не могут ему помочь, так как получаемые от них деньги и одежда в тот же день тратятся на наркотики. Принят на снабжение. Поступил на работу в пожарно-сторожевую охрану. Работает в одном месте свыше двух лет. Ценится как хороший работник, получил повышение по службе. В первые два—три месяца были срывы, в дальнейшем строго ограничивается прописанной дозой (6,0 — 1% морфия).

За недостатком места мы ограничиваемся двумя приведенными примерами. Описанные результаты снабжения мы рассматриваем как хорошие. Из 85 человек принятых на снабжение, хорошие результаты мы получили в 40,1%. Среди них— один инженер, два врача, несколько лиц среднего медперсонала. Следующую группу составляют больные, результаты снабжения которых оцениваются как удовлетворительные. Приведем для примера один случай.

Больная Е., 33 лет, из здоровой семьи. Брат неустойчивый психопат, наркоман. В 19-летнем возрасте вместе с братом попала в компанию учащейся молодежи, где из любопытства пробовали нюхать кокаин, впрыскивать морфий и т. д. Быстро привыкла (одновременно с братом) к кокаину, затем перешла на морфий. В течение 12 лет делает инъекции морфия, а при отсутствии последнего принимает внутрь опий и иноземцевы капли. В диспансер обратилась в 1929 г. Истощена, имеется несколько абсцессов. Многократно и безрезультатно лечилась стационарно. Находится на иждивении отца. Последние три года нетрудоспособна. Все жизненные интересы сосредоточены вокруг морфия. В 1930 г. принята на снабжение, которое продолжается до сих пор. Стала спокойнее, уравновешеннее. Все эти годы работает (машинистка, делопроизводитель). С работой справляется, по временам наблюдаются срывы, когда больная не ограничивается прописанной дозой, тратит часть зарплаты на нелегальные доставания наркотиков, делает прогулы. Обычно же ограничивается прописанным количеством (6,0—2% морфия в день), помогает престарелым родителям, ведет трудовой образ жизни.

Удовлетворительные результаты мы получили в 31,9%. Наконец, к последней группе принадлежат случаи, где состояние больных не изменялось к лучшему и снабжение не дало положительного эффекта.

П., 29 лет, лекпом, в 1932 году обратился к нам для лечения по поводу наркомании. В течение восьми лет вводит подкожно и внутривенно морфий или героин. Назвать дозу затрудняется, принимает столько, сколько может добыть. При дозах ниже 0,2 pro die—явления абстиненции. До сих

пор не лечился, но в связи с потерей трудоспособности за последнее время и затруднениями в добыче морфия, решил заняться лечением. Направлен в стационар, где пробыл $2^1/_2$ месяца. Рецидив в день выхода из больницы. Через несколько месяцев снова госпитализирован, но под каким-то предлогом выписался из больницы через 5—6 дней. Больной опускается все ниже в социальном отношении. Не имеет определенного места жительства и занятий. Спит летом в садах, под мостами и т. п., зимой—в ночлежке или находит приют у знакомых морфинистов. Санитарную обработку получает бесплатно „на любом санпропускном пункте". Питается исключительно хлебом и водой. Дневную порцию хлеба „зарабатывает", прося подаяние у дверей булочной, на что тратит полчаса в день, 8—10 часов в день уходит на обход аптек, где реализует ежедневно 10—15 рецептов на наркотики, набирая 02,—0,3 морфия в сутки. Рецепты выписывает сам на бланках, которые изредка крадет в амбулаториях. Других преступлений не совершает. За полтора года израсходовал всего около двух рублей. Лишившись шприца, сам сделал себе новый (резиновый баллончик из детской соски, привязанный к игле). Двухмесячный опыт снабжения больного морфием ничего не изменил в его образе жизни и состоянии. Предпринята третья, также неудачная попытка стационарного лечения, после чего больной вскоре исчез из нашего поля зрения.

Отсутствие положительных результатов мы наблюдали в 28% случаев. Интересно, что эти безрезультатные случаи почти целиком относятся к психопатам. Так, из 51 психопата, принятых на снабжение, удовлетворительные результаты были получены у 13 человек, хорошие у 17 и отсутствие результатов у 21. Между тем, из 31 „нормальных" морфинистов отсутствие результатов отмечено только в трех случаях, хороших же и удовлетворительных результатов по 14.

Рекомендуя предлагаемый метод снабжения хроников мы вместе с тем должны предостеречь от недостаточно серьезного его применения. Необходимо строго руководствоваться изложенными выше медицинскими и социальными показаниями. Не менее важен систематический и правильно поставленный контроль (обследования на дому и на производстве) и учет. Невыполнение этих правил может дискредитировать дело[1]. Само собой разумеется, что снабжение хроников наркотиками должно рассматриваться как временная, паллиативная мера, отнюдь не избавляющая от необходимости дальнейших поисков радикальных лечебных мероприятий и от повторных попыток лечения больных.

ЛИТЕРАТУРА

Marschall. Rep. of Board Health Michigan. 1878.
Earle. Chicago Med. Rev. 1880.
Brown. Amer. Jour. of Public Health. 1914.
Farr. New-York Med. Jour. 1915.
Hamilton. New-York Med. Jour. 1919.
Серейский. Ztschr. f. d. ges Neurol. u. Psych. 1925.
Голант. Проблемы морфинизма — Труды Гос. инст. медиц. знаний.
Claude. Revue Scientifique. 1923.
Meyer. Mediz. Klinik. 1924.
Sprague. Lancet-Clinic. 1907.
Wholey. Penn. Med. Jour. 1913.
Henderson. Glasgow Med. Jour. 1916.
Block. New-York Med. Jour. 1916.
Hubbard. Monthly Bull. Dep. of Health New-York, febr. 1920.
Ioël. Therap. Gegenwart. 1923.
Ioël und *Ettinger*. Arch. f. Exp. path. u. Pharmokol. 1926.
Ioël und *Fränkel*. Therap. die Gegenwart. 1926.
Они же. Klin. Wochenschr. 1925 и 1927.
Они же. Der Cocainismus. Berlin. 1924.
Иоэль. Лечение наркоманий. Изд-во „Научная мысль". 1930.
Terry and *Pellens*. The Opium Problem. New-York. 1928.
Lewin. Des Paradis artificiels. Paris. 1928.
Sollier. Sem med. 1894; Presse med. 1893 и 1905. Bull. de l'Acad. de Med. 1922.
Erlenmeyer. Die Morphiumsucht. 1883.
Он же. Neurol. u Psychiatrie. 1926.
Эрленмейер и Салье. Морфинизм и его лечение Спб. 1899.

[1] В. В. Браиловский в статье „Больше внимания наркоманической опасности" (Сов. врач. № 11—12, 1930 г.) сообщает о беспорядочном и нерациональном отпуске наркотиков, широко применявшемся в одном из медицинских учреждений Сталинграда и справедливо осуждает его.

DISPENSARY; OBSERVATIONS ABOUT MORPHINISM

By N. V. Kantorovitsch, Docent, Leningrad

Narcolcgical Department of the Nevro-Psychiatrical Dispensary and of the Bechterew Neuro-Psychiatrical Institute in Leningrad

This is a report of the six years' experiences of the author with the treatment by dispensary methods of morphinomaniacs (125 cases carefully studied). The following conclusions of the author may be emphasized here.

1. 48% of narcomaniacs, had been absolutely healthy as to their neuro-mental condition, before their disease began; the rest of the patients revealed different psychotic traits (psychasthenical, hysterical or unstable). No distinguishing characteristics in the bodily frame of narcomaniacs could be noted.

2. The chief cause of the increase in the number of morphinomania cases was the imprudent and incorrect indication of treatment by physicians.

3. Every man is liable to become morphomaniac under the influence of a prolonged intoxication with morphine. Psychopathic organisation favours the origin of the condition but it is not obligatory at all.

4. The existing methods of treatment give but a slight positive effect (only in 11,4% of cases). A great number of incurable cases makes in necessary to indicate for chronic patients the regular use of small doses of the drug. The author elaborated the indications and contraindications as also the dosage and the control for such a provision of chronic morphinomaniacs, which method of treatment gave favorable results as to the improvement of general condition and the working ability of patients.

OBSERVATIONS DE DISPENSAIRE SUR LE MORPHINISME

Par le Prof. agr. N. V. Kantorovitch (Leningrad)

Service narcologique du Dispensaire neuro-psychiatrique et de l'Institut neuro-psychiatrique de Leningrade

L'auteur parle de ces expériences de six années dans le travail thérapeutique du dispensaire parmi les morphinomanes, héroinomanes et opiophagues (125 cas). Ces conclusions peuvent être résumées ainsi:

1. Avant de tomber malades 48 p. c. des narcomanes réjouissaient d'une parfaite santé neuromentale. Les autres malades révélaient des traits psychasthéniques, hystériques ou signes d'instabilité dans leur organisation neuromentale prémorbide.

2. La cause principale de la morphinomanie était dans la plupart des cas une préscription imprudente et incorrecte du médecin.

3. Chaque homme est capable de devenir morphinomane sous l'influence d'une intoxication prolongée par la drogue. La constitution psychopathique favorise la morphinomanie, mais elle n'est indispensable pour que cet état se développe.

4. Les méthodes existantes du traitement ne donnent qu'un effet bien faible (11,4 p. C. des cas positifs). C'est pourquoi se pose la question du traitement des cas incurables. L'auteur a élaboré une méthode qui consiste à laisser les malades prendre régulièrement de petites doses de morphine auxquelles ils se sont habitués. Il en donne des indications et les contraindications dans le présent article. Or cette méthode de l'auteur a fourni des résultats favorables quant à l'amélioration de l'état général des malades chroniques et la restitution de heur capacité au travail.
